# Extracorporeal treatment for poisoning to beta-adrenergic antagonists: systematic review and recommendations from the EXTRIP workgroup

**DOI:** 10.1186/s13054-021-03585-7

**Published:** 2021-06-10

**Authors:** Josée Bouchard, Greene Shepherd, Robert S. Hoffman, Sophie Gosselin, Darren M. Roberts, Yi Li, Thomas D. Nolin, Valéry Lavergne, Marc Ghannoum, the EXTRIP workgroup, Josée Bouchard, Greene Shepherd, Robert S. Hoffman, Sophie Gosselin, Darren M. Roberts, Yi Li, Thomas D. Nolin, Valéry Lavergne, Marc Ghannoum, Badria Alhatali, Kurt Anseeuw, Steven Bird, Ingrid Berling, Timothy E Bunchman, Diane P Calello, Paul K Chin, Kent Doi, Tais Galvao, David S Goldfarb, Hossein Hassanian-Moghaddam, Lotte CG Hoegberg, Siba Kallab, Sofia Kebede, Jan T Kielstein, Andrew Lewington, Etienne M Macedo, Rob MacLaren, Bruno Megarbane, James B Mowry, Thomas D Nolin, Marlies E Ostermann, Ai Peng, Jean-Philippe Roy, Anitha Vijayan, Steven J Walsh, Anselm Wong, David M Wood, Christopher Yates

**Affiliations:** 1 Research Center, CIUSSS du Nord-de-L’île-de-Montréal, Hôpital du Sacré-Coeur de Montréal, University of Montreal, Montreal, QC Canada; 2 Division of Practice Advancement and Clinical Education, UNC Eshelman School of Pharmacy, Chapel Hill, NC USA; 3 Division of Medical Toxicology, Ronald O. Perelman Department of Emergency Medicine, NYU Grossman School of Medicine, New York, NY USA; 4 Centre Intégré de Santé et de Services Sociaux (CISSS) Montérégie-Centre Emergency Department, Hôpital Charles-Lemoyne, Greenfield Park, QC Canada; 5 Department of Emergency Medicine, McGill University, Montreal, QC Canada; 6 Centre Antipoison du Québec, Quebec, QC Canada; 7 Departments of Renal Medicine and Transplantation and Clinical Pharmacology and Toxicology, St Vincent’s Hospital, Sydney, NSW Australia; 8 St Vincent’s Clinical School, University of New South Wales, Sydney, NSW Australia; 9 Emergency Department, State Key Laboratory of Complex Severe and Rare Diseases, Peking Union Medical College Hospital, Chinese Academy of Medical Science and Peking Union Medical College, Beijing, China; 10 Department of Pharmacy and Therapeutics, and Department of Medicine Renal-Electrolyte Division, University of Pittsburgh Schools of Pharmacy and Medicine, Pittsburgh, PA USA; 11 Verdun Hospital, 4000 Lasalle Boulevard, Verdun, Montreal, QC H4G 2A3 Canada

**Keywords:** Beta-blockers, ECLS, Hemodialysis, Hemoperfusion, Overdose, Intoxication

## Abstract

**Background:**

β-adrenergic antagonists (BAAs) are used to treat cardiovascular disease such as ischemic heart disease, congestive heart failure, dysrhythmias, and hypertension. Poisoning from BAAs can lead to severe morbidity and mortality. We aimed to determine the utility of extracorporeal treatments (ECTRs) in BAAs poisoning.

**Methods:**

We conducted systematic reviews of the literature, screened studies, extracted data, and summarized findings following published EXTRIP methods.

**Results:**

A total of 76 studies (4 in vitro and 2 animal experiments, 1 pharmacokinetic simulation study, 37 pharmacokinetic studies on patients with end-stage kidney disease, and 32 case reports or case series) met inclusion criteria. Toxicokinetic or pharmacokinetic data were available on 334 patients (including 73 for atenolol, 54 for propranolol, and 17 for sotalol). For intermittent hemodialysis, atenolol, nadolol, practolol, and sotalol were assessed as dialyzable; acebutolol, bisoprolol, and metipranolol were assessed as moderately dialyzable; metoprolol and talinolol were considered slightly dialyzable; and betaxolol, carvedilol, labetalol, mepindolol, propranolol, and timolol were considered not dialyzable. Data were available for clinical analysis on 37 BAA poisoned patients (including 9 patients for atenolol, 9 for propranolol, and 9 for sotalol), and no reliable comparison between the ECTR cohort and historical controls treated with standard care alone could be performed. The EXTRIP workgroup recommends against using ECTR for patients severely poisoned with propranolol (strong recommendation, very low quality evidence). The workgroup offered no recommendation for ECTR in patients severely poisoned with atenolol or sotalol because of apparent balance of risks and benefits, except for impaired kidney function in which ECTR is suggested (weak recommendation, very low quality of evidence). Indications for ECTR in patients with impaired kidney function include refractory bradycardia and hypotension for atenolol or sotalol poisoning, and recurrent torsade de pointes for sotalol. Although other BAAs were considered dialyzable, clinical data were too limited to develop recommendations.

**Conclusions:**

BAAs have different properties affecting their removal by ECTR. The EXTRIP workgroup assessed propranolol as non-dialyzable. Atenolol and sotalol were assessed as dialyzable in patients with kidney impairment, and the workgroup suggests ECTR in patients severely poisoned with these drugs when aforementioned indications are present.

**Supplementary Information:**

The online version contains supplementary material available at 10.1186/s13054-021-03585-7.

## Introduction

Poisoning from β-adrenergic antagonists (BAAs), also referred as β-blockers, can result in bradycardia, hypotension, dysrhythmias, and cardiogenic shock. Treatment is primarily supportive, but in severe cases high-dose insulin euglycemic therapy, vasopressors, and extracorporeal life support (ECLS) may be required. Extracorporeal treatments (ECTRs) are mentioned as part of the management of BAA poisoning, although their place remains uncertain and controversial [1]. The EXtracorporeal TReatments In Poisoning (EXTRIP) workgroup is composed of international experts representing diverse specialties and professional societies (Additional file 1). Its mission is to provide recommendations on the use of ECTRs in poisoning (http://www.extrip-workgroup.org) [2–5]. We present EXTRIP’s systematic review and recommendations for the use of ECTR in patients with BAA poisoning.

### Clinical pharmacology and toxicokinetics

BAAs are among the most commonly prescribed drugs for the prevention and treatment of cardiovascular disease [6, 7]. BAAs bind to β-adrenergic receptors, thereby competitively inhibiting the binding of epinephrine and norepinephrine to these receptors, and impairing conduction and contraction. Aside from their relatively small molecular size, BAAs have considerable heterogeneity regarding their physicochemical characteristics and pharmacokinetics (Table 1). For example, labetalol, propranolol, and carvedilol have a large volume of distribution, extensive protein binding, substantial hepatic metabolism, negligible renal clearance, and do not require dose modification in chronic kidney disease (CKD), whereas sotalol, nadolol, and atenolol have opposite characteristics. Additionally, their different properties influence their clinical effect; these include selectivity to the β-1 adrenergic receptors (e.g., metoprolol > propranolol), α-adrenergic antagonist activity (e.g., carvedilol, labetalol), intrinsic sympathomimetic activity (e.g., acebutolol, pindolol), membrane-stabilizing activity (MSA) from sodium channel blockade (e.g., propranolol, acebutolol, and labetalol), central nervous system (CNS) depression (e.g., propranolol), and Class III antidysrhythmic effect because of antagonism of potassium channels (e.g., sotalol). For several commercialized BAAs, intravenous and/or sustained-release forms are available.

**Table 1:** Physicochemical properties and pharmacokinetics of immediate-release β-adrenergic antagonists

Drug	MW (Da)	Protein binding (%)	V_D_ (L/kg)		F (%)	T_MAX_ (h)	Endogenous T_1/2_ (h)		Endogenous CL (mL/min)		Renal CL (mL/min), Normal GFR	Therapeutic range (mg/L)	References
			Normal GFR	CKD			Normal GFR	ESKD	Normal GFR	ESKD			
Acebutolol	336	10–25	1.5–2.5	N/A	35–50	2.0–4.0	4–10&		600–800	N/A	150–300	0.2–2	[19, 68, 69, 80, 138–148]
Alprenolol	249	80–90	2.5–3.5	N/A	5–15	1.0–2.0	2–4	N/A	800–1000	N/A	50	0.03–0.15	[149–152] [153]
Atenolol	266	0–5	1.0–1.2		50–60	3.0–3.5	5–8	50–100	140–180	20	120–140	0.1–1.5	[11, 20, 73, 78, 79, 85, 90, 105, 118, 124, 148, 154–163]
Betaxolol	344	50	4.5–6.0	5.0–6.5	75–90	2.5–4.0	14–16	25–35	220–270	100–150	50	0.005–0.05	[86, 148, 164–168]
Bisoprolol	325	30	2.0–3.0		90	1.5–2.5	9–12	25–35	200–250	50	120–150	0.01–0.1	[93, 98, 148, 169–175]
Bopindolol	381	N/A	1.8–2.0	N/A	70	1.0–2.0	4–6	8	350–400	N/A	N/A	0.001–0.015	[148, 176–180]
Carteolol	292	10–30	4	N/A	85	2.0	5–7	30–35	650	N/A	250	0.01–0.1	[148, 181–183]
Carvedilol	405	98	1.5–2.5	N/A	20–30	1.0–3.0	6–7		600		5	0.02–0.2	[96, 148, 184–190]
Celiprolol	379	25	4–5	N/A	30–70	2.0–4.0	5–7	N/A	900–1000	N/A	180–220	0.05–0.5	[191–193]7 [148, 194–200]
Cetamolol	310	N/A	3.5	2.5	N/A	2.5–3.0	7	10–12	420*	150	100–150	0.01–0.1	[201–203]
Esmolol	295	55	2.0–3.5		Not applicable		0.2		10,000–15,000		100–200	0.15–2	[95, 148, 204, 205]
Labetalol	328	50	5.0–9.0		20–30	0.5–1.5	3–10	10–12	1200–2000		20	0.03–0.3	[92, 206–212] [94, 148, 213]
Medroxalol	372	N/A	10–15	N/A	30–50	2–3	7–15	N/A	1000–1100	N/A	80–100	N/A	[213–215]
Mepindolol	262	55	5.7**	N/A	N/A	1.4	3–6		650**	N/A	0**	0.007–0.07	[88, 148, 216, 217]
Metipranolol	309	70	3–4	N/A	40–50	0.5–2.0	2.5–3.0		1100–1300	N/A	120–150	0.02–0.1	[97, 148, 218–221]
Metoprolol	267	10	3.0–4.0	N/A	40–60	1.5–2.0	3–5		800–1200		100	0.03–0.5	[222–225] [9, 73, 82, 106, 148, 226–230]
Nadolol	309	15–25	1.5–2.0	N/A	30	2.8	10–15	30–45	120–250	30	80–120	0.01–0.25	[75, 89, 148, 231–234]
Nebivolol	405	98	9–12		Variable	1–3	10–15		800–1000		30	0.001–0.05	[148, 235–241]
Oxprenolol	265	80–85	0.8–1.2	0.8	35–50	0.5–1.5	1–2		600–750	550	10	0.05–0.3	[84, 148, 242–252]
Penbutolol	291	90–95	0.5–1.0	N/A	90	1.0–2.0	15–20	30	300–600	N/A	5	0.01–0.3	[253] [148, 254–262]
Pindolol	248	40–55	1.3–2.3	1.6–1.8	50–90	0.5–1.5	3–5		450–550	180–240	150–250	0.02–0.15	[148, 263–269]
Practolol	266	57	1.5	N/A	90–100	2–5	10–13	60–80	135	20	100–120	1.5–5	[65, 67, 148, 270–274]
Prenalterol	225	5	2.5–3.5	N/A	25–35	0.5–2.5	1.5–2.5	N/A	800–1400	N/A	200–800	0.01–0.04	[275–282]
Propranolol	259	85–95	3.0–5.0		20–50	1.5–2.0	3–5		800–1200		5	0.02–0.3	[20, 66, 70, 74, 154, 230, 283–300] [73, 87, 131, 148, 301, 302]
Sotalol	272	0	1.3–1.5		90	2.5–3.5	5–9	35–60	120–160	20–25	80–120	0.5–3	[71, 83, 303–307] [13, 15, 115, 148, 303, 306, 308–310]
Talinolol	363	60**	3.0–3.5		55	2.5–3.5	10–12	20–25	320–380		150–200	0.04–0.15	[99, 104, 107, 148, 311–313]
Timolol	316	10	2.0–2.5	N/A	60	1.3–2.0	3–5		450–580	N/A	100	0.005–0.1	[300, 314–317] [72, 148, 315]
Tolamolol	316	90	1.2–1.8	N/A	N/A	1–3	2–3		1100	N/A	N/A	N/A	[318–320]

MW: Molecular weight, V_D_: Volume of distribution, F: bioavailability, T_MAX_: Time to maximum concentration, T_1/2_: elimination half-life, CL: Clearance, N/A: Not available

*  Not adjusted for bioavailability, ** No reference from primary data (taken from reviews)

& conflicting data, perhaps due to non-sensitive assays which included measurement of metabolites in early reports [68, 69]

Total body clearance and volume of distribution were obtained from intravenous data. If these data were unavailable but reported for oral data (i.e., as V/F or CL/F), then values were adjusted for bioavailability

This systematic review has taken the liberty to review all BAAs for which data exist, even if some are not currently commercially available

In overdose, a prolonged absorption phase, saturation of enzymatic biotransformation, and poison-induced impairment of blood flow to organs may all contribute to a prolonged apparent elimination half-life, which has been described for propranolol [8], metoprolol [9, 10], atenolol [11], and sotalol [12–14] although this finding is inconsistent [15–18]. Protein binding does not appear to be modified in supratherapeutic concentrations [19, 20].

### Overview of toxicity

Over the last 5 years, the number of BAA exposures reported to the United States National Poison Data System has increased [21], and is associated with 3.9% of fatal poisonings [21]. In 2019, 11,166 single ingredient BAA exposures were reported in the US including 19 fatalities [21]. Manifestations of BAA poisoning range from asymptomatic bradycardia to cardiogenic shock and death [22–25]. Cardiovascular symptoms usually appear within 2 h of ingestion and are unlikely to occur in an asymptomatic patient after 6 h from ingestion for immediate-release formulations [22, 26, 27], 8 h for sustained-release formulations, and 12 h for sotalol [12, 23, 24, 28]. Decreased consciousness and bronchospasm may occur after these periods, even with normal blood pressure and electrocardiogram [26, 29]. Other manifestations of poisoning from BAAs include hyperkalemia and hypoglycemia [30, 31]. Highly lipophilic drugs, like propranolol, penetrate the blood–brain barrier causing delirium, coma, and seizures [27, 30, 32, 33]. Sotalol, which also possesses potassium efflux channel blocking properties, causes QT interval prolongation and severe ventricular dysrhythmias, including torsade de pointes [12, 32, 34, 35]. In overdose, receptor selectivity is lost, leading to overlapping manifestations among BAAs [36, 37].

Some publications report a linear or threshold relationship between dose and outcome [32, 37]. For specific BAAs, a positive correlation was noted for propranolol [27, 32, 36], sotalol [32], atenolol [32], metoprolol [32, 38], carvedilol [39], and talinolol [36]. Unintentional exposures and inadvertent ingestions in young children rarely cause severe toxicity due to the smaller doses involved, although exceptions are reported [40, 41]. Quantification assays for BAAs are rarely available to guide clinical decisions, and concentrations correlate poorly with the development of symptoms [42–44], except for sotalol [45–48].

Fatalities from BAA ingestions are more likely if co-ingested with cardioactive drugs, such as calcium channel blockers [22, 32, 37, 49]. In cohorts of severe BAA poisoning, reported mortality rates range between 0 and 13% [21–23, 25, 32, 50–52].

Management of BAA poisoning is primarily supportive [1]. Although outside the scope of this review, standard care includes gastrointestinal decontamination, atropine, inotropes and vasopressors, temporary cardiac pacing, glucagon, intravenous calcium, high-dose euglycemic hyperinsulinemia, and extracorporeal life support (ECLS) [1, 53–57].

## Methods

The workgroup developed recommendations following the EXTRIP methodology previously published [3] with modifications, updates, and clarifications. PRISMA statement was followed for reporting items of the presented systematic review of the literature. The full methods are presented in the online Additional file 1.

The search strategy used was as follows: [(dialysis or hemodialysis or haemodialysis or hemoperfusion or haemoperfusion or plasmapheresis or plasmaphaeresis or hemofiltration or haemofiltration or hemodiafiltration or haemodiafiltration or plasma exchange or CRRT or CVV* or CKRT or exchange transfusion) and (beta blocke* or beta-adrenergic or acebutolol or alprenolol or atenolol or betaxolol or bisoprolol or bopindol or carteolol or carvedilol or celiprolol or cetamolol or esmolol or labetalol or medroxalol or mepindol or metipranolol or metoprolol or nadolol or nebivolol or oxprenolol or penbutolol or pindolol or practolol or prenalterol or propranolol or sotalol or talindolol or talinolol or timolol or tolamolol)].

## Results

**Fig. 1:**
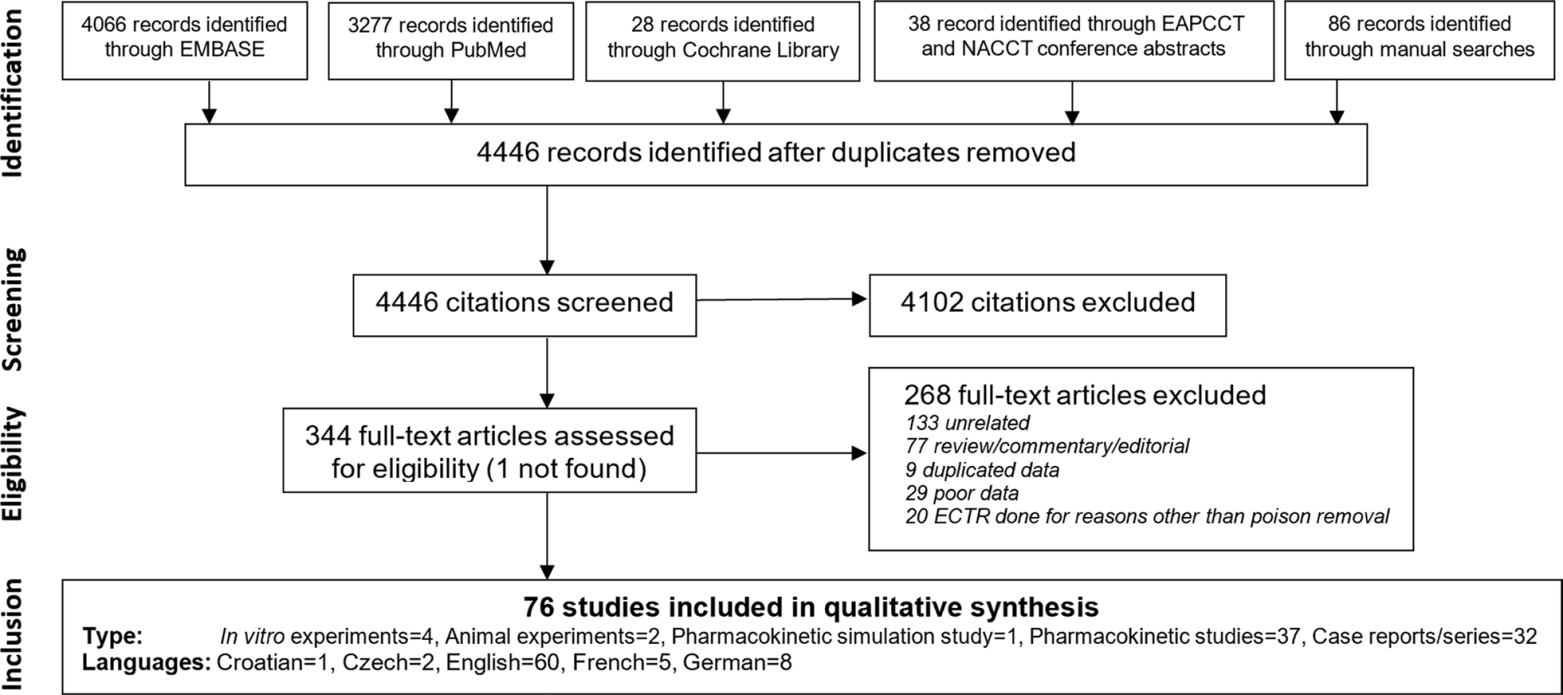
Process of selection and inclusion of studies in the review

Results of the literature search are presented in Fig. 1.

In the final analysis, 76 studies were included for qualitative analysis, including 4 in vitro experiments [58–61], 2 animal experiments [62, 63], 1 pharmacokinetic simulation study [64], 37 pharmacokinetic studies [65–101], and 32 case reports/series [13, 15, 35, 102–130]. No comparative studies or randomized controlled trials were identified.

### Summary of evidence

#### Dialyzability

Because of the large heterogeneity in BAAs pharmacokinetics, no a priori overall estimation of dialyzability can be generalized for this entire drug class. Half-lives and clearances of BAAs obtained during ECTR are summarized in Table 2. Pharmacokinetic or toxicokinetic data related to ECTR were available for a total of 334 patients. Ninety percent of the pharmacokinetic articles were published prior to 1992. Although these older reports had robust methods, with several subjects and serial samplings of BAAs concentrations in blood and dialysate, they must be interpreted with caution as they may not reflect current hemodialysis technology. With improved blood and effluent flows and better catheters and filters, these data are expected to be more favorable. For example, atenolol clearance by ECTR has tripled in 30 years [78, 101], bisoprolol clearance has doubled in 20 years [98, 101], and nadolol clearance has increased by 50% in 5 years [75, 89].

**Table 2:** Half-life and clearance of β-adrenergic antagonists during extracorporeal treatments

Drug	ECTR	T_1/2_ (Hours)					Clearance (mL/min)					References
		During ECTR			Endogenous		ECTR			Endogenous		
		Median	n	Range	Normal GFR	ESKD	Median	n	Range	Normal GFR	ESKD	
Acebutolol	HD	6.1 (Met = 7.2)	7	2.2–7	4–10		45 (Met = 33.6)	12	30.5–55.1	600–800	N/A	[68, 69, 80, 109, 113]
	HF-HP	0.3	1				151	1				
Atenolol	HD	4.6	48	0.5–17.8	5–8	50–100	119.5	25	18–311	140–180	20	[73, 76, 78, 79, 85, 90, 91, 100, 101, 105, 118, 123, 124, 128]
	CKRT	22	2	14.5–29.5			47	3	19.9–48			
	HD-HP	3.4	1				N/A					
	PD	23	17	19.2–34			2.6	7	1.3–3.7			
Betaxolol	HD	N/A			14–16	25–35	17.5 ± 0.7	12		220–270	100–150	[86]
	PD	N/A					11.7 ± 1.2	12				
Bisoprolol	HD	7.8	16	6.4–9.6	9–12	25–35	41.8	16	30.5–70	200–250	50	[93, 98, 101]
	PD	23.6	3	20.8–26.4			N/A					
Carvedilol	HD	4.6	8	4.1–5.3	6–7		12.1	14	12.1–38.6	600		[96, 101]
Esmolol	HD	0.12 ± 0.1	6		0.2		Met = 76.8 ± 39.1	6		10,000–15,000		[95]
	PD	0.13 ± 0.1	6				Met = 2.7 ± 0.5	6				
Labetalol	HD	1.8	7	0.6–3.8	3–10	10–12	37.4	14	25.7–97	1200–2000		[92, 94]
	PD	13.1 ± 6.3	8				1.9 ± 1.7	8				
Mepindolol	HD	3.0	2		3–6		31	2		650	N/A	[88]
Metipranolol	HD	1.4 ± 0.5	8		2.5–3.0		N/A			1100–1300	N/A	[97]
Metoprolol	HD	2.9 (Met = 5)	8	2.3–3	3–5		101	8		800–1200		[73, 101, 106]
	HP	2.2	1				96.1	1				
Nadolol	HD	3.5	6	3.0–8.5	10–15	30–45	82	15	46.4–102	120–250	30	[75, 89]
Oxprenolol	HD	Met = 5.6	3		1–2		N/A			600–750	550	[84]
Practolol	HD	14 (Met = 5)	14	8–30	10–13	60–80	> 100*	6		135	20	[65, 67]
Propranolol	HP	5.6	3	4.9–8.9	3–5		189	2	188–191	800–1200		[66, 70, 73, 74, 87, 102]
	HD	3.4	23	1.5–8			9.4	11	3.8–26.5			
	TPE	1.2	1				N/A					
Sotalol	HD	7	9	3.5–9.5	5–9	35–60	80	3	67–80	120–160	20–25	[13, 15, 71, 83, 108, 115, 122]
	HP-HD	2.8	1				N/A					
	CKRT	18	1				53.1	1				
Talinolol	HD	N/A			10–12	20–25	28	7		320–380		[99, 104, 107]
	HP	3.3	2	2.7–3.8			109	2	96–121			
Timolol	HD	3.8	2	2.3–5.2	3–5		N/A			450–580	N/A	[72]

Legend: HD, Hemodialysis; HP, Hemoperfusion; HF, Hemofiltration; TPE, therapeutic plasma exchange; PD, Peritoneal dialysis; CKRT, Continuous kidney replacement therapy; ECTR, Extracorporeal treatment; ESKD, End-stage kidney disease; Met, metabolite; GFR, Glomerular filtration rate; N/A, Not available; n, number

In order to make data consistent and comparable for analysis, some transformations were performed (if necessary): half-lives were calculated graphically; clearances were calculated from removal data; when both clearance from arterio-venous differences and dialysate collections were provided, these were averaged; in some cases, clearance reported by some authors were calculated by using blood flows and plasma concentrations which may lead to overestimations [13, 98, 109], and so were recalculated

^*^Reported dialysate flow assumed to be > 300 mL/min

When measured from dialysate collection, the amount of BAA removed divided by the reported ingested dose during hemodialysis (when adjusted for a 6-h treatment and bioavailability) was 24% for atenolol [101], 18% for bisoprolol [101], ≈0% for carvedilol [101], 0.5% for labetalol [92], 3.3% for metoprolol [101], 50% for practolol [67], ≈0% for propranolol [70], and 4.6% for talinolol [99].

Data for continuous kidney replacement therapy (CKRT) are sparse: in 3 cases of atenolol overdose, CKRT removed between 8 and 25% of total body burden adjusted for a 6-h period [120, 123, 128], with atenolol clearance ranging from 20 to 48 mL/min. In one sotalol overdose, CKRT clearance was estimated as 53 mL/min [122]. These clearances are considerably inferior to those achievable during high-efficiency intermittent hemodialysis (Table 2). There is limited evidence for hemoperfusion and therapeutic plasma exchange (TPE), which can remove BAAs with extensive protein binding. This appears true for propranolol in vitro [59, 61] and in vivo [102, 131], although its high volume of distribution and high hepatic clearance substantially limit its dialyzability. Hemoperfusion in 2 patients with talinolol overdoses yielded clearances of 100–120 mL/min [104, 107] but this represented < 20% of ingested dose, due to its large volume of distribution. As for penbutolol, in vitro data show little to no effect from hemoperfusion and only a minor and slow effect from TPE [60]. For BAAs with limited protein binding, hemoperfusion would not be expected to surpass diffusive or convective techniques as confirmed in one case of metoprolol overdose in which measured clearance [106] was comparable to that obtained during hemodialysis [101]. As expected, dialyzability of BAAs by peritoneal dialysis was consistently poor, with inconsequential impact on pharmacokinetics, i.e., approximately 6% of atenolol was removed in 24 h [90], 0.1% of labetalol in 72 h [92], and the peritoneal clearance of betaxolol only represented 7.5% of total clearance [86].

An increase in serum/blood concentrations was often observed following ECTR, often referred as “rebound,” in both pharmacokinetic studies [67, 76, 83, 92] and toxicokinetic reports [13, 15, 105, 107, 115, 118, 124]. The median increase in concentration was 15% and occurred independently of volume of distribution.

Table 3 presents grading of dialyzability with the level of evidence, as defined by EXTRIP criteria (Additional file 1). The grading and level of evidence for hemodialysis was assessed as: *Dialyzable* for atenolol, nadolol, practolol, and sotalol; *Moderately dialyzable* for acebutolol, bisoprolol, and metipranolol; *Slightly dialyzable* for metoprolol and talinolol; *Not dialyzable* for betaxolol, carvedilol, labetalol, mepindolol, propranolol, and timolol. Some publications report that metoprolol may be dialyzable based on achievable clearance of 80–120 mL/min [101]. However, this only represents a small proportion of total body clearance (regardless of genetic polymorphism of clearance pathways), resulting in removal of < 10% of an ingested dose. Because of its high endogenous clearance and volume of distribution, propranolol will not be removed meaningfully by ECTR modalities.

**Table 3:** Final toxicokinetic grading according to EXTRIP criteria

Drug	PK/TK grading	Number of patients						Final grading and level of evidence
		HD	PD	CKRT	HP	TPE	HP-HD	
Acebutolol	Dialyzable	1, MET = 2						HD: Moderately dialyzable, D* HD (MET): Moderately dialyzable, C
	Moderately dialyzable	1, MET = 1						
	Slightly dialyzable	1, MET = 1						
	Not dialyzable							
Atenolol	Dialyzable	24						HD: Dialyzable, A CKRT: Slightly dialyzable, C HD-HP: Moderately dialyzable, D PD: Not dialyzable, B
	Moderately dialyzable	1		1			1	
	Slightly dialyzable			2				
	Not dialyzable		7					
Betaxolol	Dialyzable							HD: Not dialyzable, B PD: Not dialyzable, C
	Moderately dialyzable							
	Slightly dialyzable							
	Not dialyzable	12	6					
Bisoprolol	Dialyzable	5						HD: Moderately dialyzable, B
	Moderately dialyzable	14						
	Slightly dialyzable							
	Not dialyzable							
Carvedilol	Dialyzable							HD: Not dialyzable, B
	Moderately dialyzable							
	Slightly dialyzable							
	Not dialyzable	8						
Labetalol	Dialyzable							HD: Not dialyzable, B PD: Not dialyzable, C
	Moderately dialyzable							
	Slightly dialyzable							
	Not dialyzable	17	8					
Mepindolol	Dialyzable							HD: Not dialyzable, C
	Moderately dialyzable							
	Slightly dialyzable	1						
	Not dialyzable	1						
Metipranolol	Dialyzable							HD: Moderately dialyzable, C
	Moderately dialyzable	4						
	Slightly dialyzable							
	Not dialyzable							
Metoprolol	Dialyzable	M = 2						HD: Slightly dialyzable, B HD (MET): dialyzable, C HP: Slightly dialyzable, D (Normal GFR)
	Moderately dialyzable							
	Slightly dialyzable	8			1 (Normal GFR)			
	Not dialyzable							
Nadolol	Dialyzable	6						HD: Dialyzable, B
	Moderately dialyzable							
	Slightly dialyzable							
	Not dialyzable							
Oxprenolol	Dialyzable	MET = 3						HD (MET): Dialyzable, C
	Moderately dialyzable							
	Slightly dialyzable							
	Not dialyzable							
Practolol	Dialyzable	14						HD: Dialyzable, B
	Moderately dialyzable							
	Slightly dialyzable							
	Not dialyzable							
Propranolol	Dialyzable	1, MET = 2						HD: Not dialyzable, A HD (MET): Dialyzable, C HP: Slightly dialyzable, D (Normal GFR)
	Moderately dialyzable	2				1**		
	Slightly dialyzable				2 (Normal GFR)			
	Not dialyzable	13						
Sotalol	Dialyzable	6, 1 (Normal GFR)						HD: Dialyzable, B HD: Dialyzable, D (Normal GFR) CKRT: Slightly dialyzable, D HD-HP: Moderately dialyzable, D (Normal GFR)
	Moderately dialyzable	1					1 (Normal GFR)	
	Slightly dialyzable			1				
	Not dialyzable							
Talinolol	Dialyzable							HD: Slightly dialyzable, B HP: Slightly dialyzable, C (Normal GFR)
	Moderately dialyzable							
	Slightly dialyzable	8			2 (Normal GFR)			
	Not dialyzable							
Timolol	Dialyzable							HD: Not dialyzable, D
	Moderately dialyzable							
	Slightly dialyzable	1						
	Not dialyzable	1						

MET, Metabolites; PK, Pharmacokinetics TK, Toxicokinetics; HD, Hemodialysis; HP, Hemoperfusion; PD, Peritoneal dialysis; CKRT, Continuous kidney replacement therapy; TPE, Therapeutic plasma exchange; HD-HP, hemodialysis and hemoperfusion in series; GFR, Glomerular filtration rate

^*^6 additional patients would be rated as “dialyzable” but the assay was non-specific and likely measured parent drug and metabolites, so the result is uninterpretable [69]

^**^Based on half-life comparison, a criterion considered unreliable for poisons with a high Vd like propranolol, so not graded

**Fig. 2:**
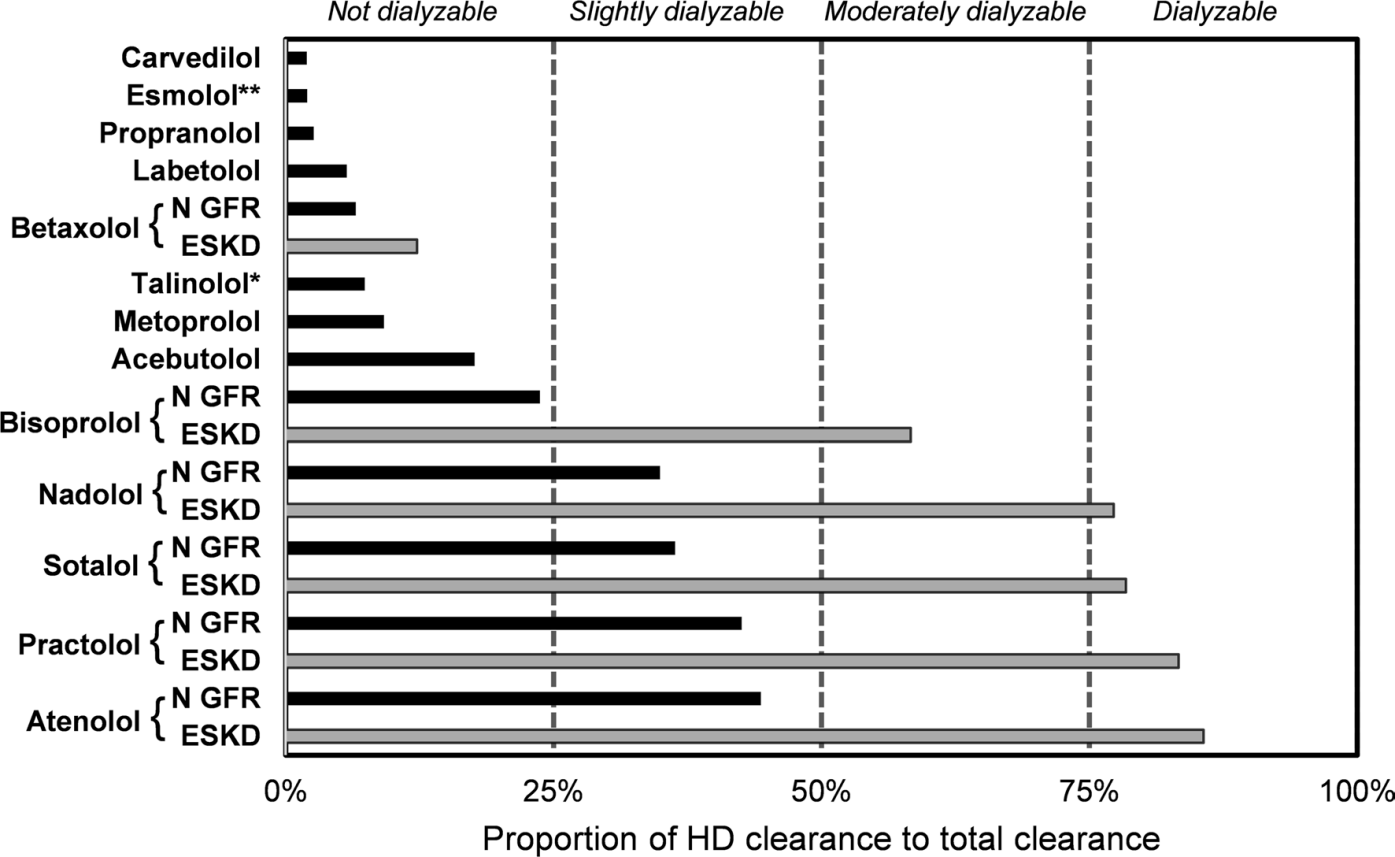
Proportion of hemodialysis clearance relative to total clearance. Legend: GFR: Glomerular filtration rate, ESKD: End-stage kidney disease, HD: Hemodialysis. *These are conservative estimations, as ECTR clearances would likely be higher if performed today. **based on endogenous clearance of 12,000 mL/min. For carvedilol, esmolol, propranolol, labetalol, talinolol, metoprolol, and acebutolol, it is assumed the ratio would apply regardless of kidney function

Although extracorporeal clearance of BAAs is independent of kidney function, its relative impact compared to total body clearance will increase for some BAAs as kidney function declines. This can be illustrated graphically (Fig. 2): for example, a hemodialysis clearance of 120 mL/min will represent 46% of total clearance for atenolol in a patient with normal kidney function (endogenous clearance = 140 mL/min) compared to 86% in an anuric patient (endogenous clearance 20 mL/min). Comparatively, ECTR clearance will have very little impact on enhancing total clearance of propranolol, regardless of kidney function. These estimates are considered conservative for several BAAs including sotalol, practolol, nadolol, and betaxolol, as the ECTR data are at least 30 years old [67, 83, 86, 89]. Limited data exist for esmolol but even when assuming an optimal hemodialysis plasmatic clearance of 300 mL/min, this would represent less than 2% of total clearance [95].

Only 7 patients that could be assessed for dialyzability grading had normal kidney function, and only two reports were identified for a BAA (sotalol) whose grading may differ depending on kidney function [13, 15]. For these two cases, dialyzability was assessed as “*Dialyzable*” for one case of hemodialysis and “*Moderately dialyzable*” for one case of hemoperfusion-hemodialysis in series.

#### Clinical data

Among case reports and case series, the panel acknowledged variability in methodological quality and lack of reporting of critical information [132]. The evidence for a clinical effect of ECTR in BAA poisoning was available for 37 patients (acebutolol = 4, atenolol = 9, carvedilol = 1, metoprolol = 1, propranolol = 9, sotalol = 9, talinolol = 4), 16 of which had impaired kidney function (Table 4). All included patients were self-poisoned, except 6 dosing errors in end-stage kidney disease (ESKD) (atenolol = 1, sotalol = 5). Bradycardia and hypotension requiring vasopressors and/or inotropes were ubiquitous features for all BAAs except for propranolol and sotalol (predominant features for sotalol were ventricular dysrhythmias).

**Table 4:** Summary of clinical findings of patients receiving extracorporeal treatments for β-adrenergic antagonist removal

	Acebutolol (n = 4)	Atenolol (n = 9)	Carvedilol (n = 1)	Metoprolol (n = 1)	Propranolol (n = 9)	Sotalol (n = 9)	Talinolol (n = 4)
*Patient characteristics*							
Age, years	20 (17–27)	45 (28–74)	21	49	31 (15–49)	66 (44–78)	21 (20–47)
Men, %	0	56	0	0	44	78	50
ESKD, %	0	11	0	0	0	44	0
*Poisoning information*							
Intentional overdose, %	100	89	100	100	100	44	100
Dose if acute ingestion, g	8.4 (4.8–12)	4.5 (2.5–10)	1.8	0.5	3.1 (0.6–5.0)	8.0 (7.2–14.4)	2.5 (1.5–5.0)
Peak concentration, mg/L	14 (10–18)	14 (2.5–70)	0.6	2.8	1.5 (0.04–3)	17 (2.5–65)	5.5 (5.0–6.1)
Time from ingestion to admission, hours	2 (2–2)	6.5 (2–8)		0.8	2 (1–8)	2.5 (1–4)	6 (2–8)
*Signs/ Symptoms / Labs*							
Coma, %	100	89	100	100	50	100	75
Altered consciousness, %	100	100	100	100	83	100	75
Bradycardia, %	100	100	100	100	100	50	100
Severe dysrhythmia, %	25	0	100	0	33	100	25
Hypotension, %	100	100	100	100	75	89	100
QRS complex duration, msec	260	128 (98–160)	N/A	N/A	104	120 (104–140)	420
Prolonged QRS complex duration, %	100	43	0	0	N/A	25	50
QT interval duration, msec	N/A	440 (400–448)	N/A	N/A	N/A	618 (509–880)	440
Prolonged QT interval, %	N/A	17	N/A	N/A	N/A	100	25
Acute kidney injury, %	100	87.5	100	0	0	38	0
Serum glucose, mmol/L	14.7	7.7 (2.2–19.2)	8.3	N/A	10.7	4.4 (1.4–7.4)	N/A
Serum bicarbonate, mmol/L	16	19 (10.8–21)	N/A	N/A	20 (15–25)	17	N/A
Serum lactate, mmol/L	1.9	4.6 (1.8–9.3)	4.7	N/A	7.6 (1.9–13.2)	1.9	N/A
Serum potassium, mmol/L	3.2	4.3 (< 0.8–8.5)	5.9	N/A	4.2 (3.7–4.7)	5.1 (3.8–7.1)	N/A
*Other treatments*							
Gastric lavage, %	25	44	0	0	67	22	75
Activated charcoal, %	75	56	0	100	50	11	25
Vasopressors/ inotropes, %	100	100	100	100	50	75	75
Mechanical ventilation, %	100	100	0	100	17	75	75
Atropine, %	100	56	0	100	67	22	25
Lipid emulsion, %	0	11	0	0	16	0	0
Pacemaker, %	100	44	100	100	33	88	50
High-dose insulin euglycemic therapy, %	0	67	0	0	33	0	0
Glucagon, %	75	100	100	100	83	33	25
Extracorporeal life support (ECLS), %	25	22	100	0	0	0	0
*Extracorporeal treatments*							
Hemodialysis, n	1	3	0	0	0	6	0
TPE, n	0	0	1	0	2	0	0
CKRT, n	0	3	0	0	0	1	0
More than 1 ECTR, n	0	2	0	0	0	0	0
HF-HP, n	1	0	0	0	0	0	0
HD-HP, n	1	1	0	0	3	1	1
HP, n	1	0	0	1	4	0	3
*Outcome*							
Death, %	0	11	0	0	11	11	50
Sequelae, %	25	11	0	0	N/A	11	N/A
Length of stay, days	30 (7–49)	22 (12–32)	23	N/A	6 (5–32)	20	N/A
Length of ICU stay, days	2 (2–2)	9.5 (1.5–28)	8	3	8.5 (4–13)	3 (2–6)	N/A
Length of life-threatening dysrhythmia	N/A	N/A	N/A	N/A	56	16 (12–120)	N/A
Length of prolonged QT interval, msec	N/A	N/A	N/A	N/A	N/A	37 (30–120)	N/A
Length of bradycardia/hypotension, hours	25 (24–26)	48 (20–168)	120	18	67 (24–70)	36	9

Results presented as medians and range. No range is presented when the number of values is one. When specific data was not reported, this was not included in the incidence

ESKD, end-stage kidney disease; TPE, therapeutic plasma exchange; CKRT, continuous renal replacement therapy; ECTR, extracorporeal treatment; HF-HP, hemofiltration-hemoperfusion; HD-HP, hemodialysis and hemoperfusion in series; HP, hemoperfusion; ICU, intensive care unit; N/A, Not available

As reflected by changing trends in the management of BAA poisoning over almost 40 years, treatments were very heterogeneous. In particular, only eight patients received high-dose insulin euglycemic therapy and four patients received ECLS, treatments now considered likely to improve outcome [1]. For these reasons, it was difficult to determine a benefit from ECTR. Three patients died of cardiogenic shock [102, 103, 108], one of irreversible brain injury [107], and one of multiorgan failure after four weeks, despite marked improvement post-ECTR [105]. The overall mortality for the cohort was 13.5%.

For sotalol, resolution of dysrhythmias/torsade de pointes was rapid with intermittent hemodialysis, often occurring during or just after treatment [13, 15, 35, 115, 116, 121], while this was more protracted with slower techniques like peritoneal dialysis (PD) [114] or CKRT [122]. For atenolol (n = 9), when hemodialysis was used, an increase in blood pressure was noted after the first treatment, with one exception [129]. Again, apparent improvement was slower with CKRT [120, 127, 128]. Dysrhythmias recurred in two patients, within two hours of ECTR cessation, requiring another session [13, 15]. Although nine patients were reported for propranolol, the clinical impact of ECTR could only be analyzed in two patients: one improved slowly after hemoperfusion [125] while the other improved after TPE but had recurrence of hypotension shortly after [130]. For acebutolol, four patients were described, three of which improved during ECTR [109, 113, 117], while this was uncertain in one patient who received hemoperfusion [112]. In all four patients of talinolol poisoning, hemoperfusion was employed alone or in combination with hemodialysis, and two of them died [103, 107]. There was only one patient described for carvedilol [126] and metoprolol [106], which were difficult to interpret because of the co-ingested calcium channel blockers in both cases. No ECTR-associated complications were described in the cohort.

In summary, clinical improvement from ECTR was generally noted with BAAs considered dialyzable such as atenolol and sotalol when high-efficiency ECTRs were used, whereas this was questionable with other BAAs or when techniques with lower efficiency were used.

To further measure the effect of ECTR, outcomes of the ECTR cohort were compared to historical controls not receiving ECTRs (Table 5). Unfortunately, this analysis is severely hampered by the small numbers of reported patients, the variability in treatments provided and the heterogeneity of populations compared. For example, historical controls reported to poison control centers are expected to have more benign features than those included in the ECTR cohort. Overall, the mortality of patients receiving ECTRs for BAA poisoning was greater than those reported in historical controls, including one cohort of critically ill patients [23]. Aside from mortality, the only outcome that could be compared to assess the benefit of ECTR was the median duration of QT interval prolongation in sotalol poisoning, which was 37 h [IQR 33.5, 78.5] for the ECTR cohort (median maximal QTc interval = 140% of normal) versus 75 h [IQR 57, 87.5] in one historical cohort (median maximal QTc interval = 172%) [12]. However, this analysis is underpowered. With regard to harms and costs, the use of ECTR is associated with an increased risk of catheter- and ECTR-related complications and added costs which will vary depending on the choice of technique and the geographical location [133]. It is possible that ECTR may exacerbate hypotension in some cases despite the absence of net ultrafiltration, although the incidence of this risk and its magnitude are unknown.

**Table 5:** Extracorporeal treatments + standard care versus standard care in β-adrenergic antagonists poisoning (evidence profile table)

Quality assessment							Summary of findings				Importance
Drug	Study design	Risk of bias	Inconsistency	Indirectness	Imprecision	Other considerations	ECTR + standard care	Standard care (controls)	Impact	Quality	
Mortality											
All β-adrenergic antagonists^a^ n = 10	Observational studies	Very serious^b^	Not serious	Serious ^c^	Serious ^d^	Publication bias strongly suspected ^e^	13.5% (5/37)	ICU data 8.2% (9/110) admitted in 1 ICU 2002–9 [23] PCC and hospital data 3.8% (63/1678) 0/858: German PCC 2001–11 single-substance [32] 1/11: children, self-harm [50] 2/73:1 hospital 1993–7 [37] 0/40: 1 hospital 1966–80 [25] 4/280: 2 PCCs 1992–8, [22] 60/416: US PCCs 2017–19, at least major effect [21, 51, 52]	Comparable mortality between the ECTR group and the control group admitted to ICU (risk difference = 53 more deaths per 1000 patients in the ECTR group (with a 95% CI from 68 less to 175 more deaths per 1000)	⨁◯◯◯ VERY LOW	CRITICAL
Propranolol^f^ n = 5	Observational studies	Very serious^b^	Not serious	Serious ^c^	Serious ^d^	Publication bias strongly suspected ^e^	11.1% median dose 3.1 g (1/9)	Ranging from 0 to 2.1% 0/41 German PCC 2001–11 single substance median dose 0.4–0.5 g [32] 7/339 UK PCC 2017–18, median dose 0.6 g [321] 0/50: 1 hospital 1993–7 mean dose 1.3 g [37] 0/18 1979–1985 mean dose 1.6 g [36]	Groups not comparable	⨁◯◯◯ VERY LOW	CRITICAL
Sotalol^g^ n = 3	Observational studies	Very serious^b^	Not serious	Serious ^c^	Serious ^d^	Publication bias strongly suspected ^e^	11.1% median 8.0 g (1/9)	Overall = 0% 0/31: German PCC 2001–11 single substance [32] 0/6: Case in Finland 1977–1980, mean dose 5.7 g [12]	Groups not comparable	⨁◯◯◯ VERY LOW	CRITICAL
Atenolol^h^ n = 3	Observational studies	Very serious^b^	Not serious	Serious ^c^	Serious ^d^	Publication bias strongly suspected ^e^	11.1% median 4.5 g (1/9)	Overall = 0% 0/48: German PCC 2001–11, single substance, median dose 0.5–0.8 g [32] 0/10: 1 hospital 1993–7 mean dose 2.0 g [37]	Groups not comparable	⨁◯◯◯ VERY LOW	CRITICAL
Duration of QT interval prolongation											
Sotalol^i^ n = 4	Observational studies	Very serious^b^	Not serious	Serious ^c^	Serious ^d^	Publication bias strongly suspected ^e^	Median = 37 h [33.5, 78.5] 3 pts, median 8 g	Median = 75 h [57, 87.5] 6 pts median dose 6.2 g 1977–80 [12]	No formal comparison possible due to the small sample size of the ECTR group	⨁◯◯◯ VERY LOW	IMPORTANT
Serious complications of catheter insertion ^j^											
n = 5 ^k^	Observational studies	Not serious	Not serious ^l^	Not serious ^m^	Not serious ^n^	Strong association ^o^	Rate of serious complications of catheter insertion varies from 0.1% to 2.1%	≈ 0	Absolute effect is estimated to be varying from 1 to 21 more serious complications per 1000 patients in the ECTR group	⨁⨁⨁◯ MODERATE	CRITICAL
Serious complications of ECTR ^p^											
n = 4^q^	Observational studies	Not serious	Not serious	Not serious	Not serious	Strong association ^r^	Rate of serious complications of ECTR varies according to the type of ECTR performed from 0.005% (IHD and CKRT), to 0.6% (TPE) and up to 1.9% (HP)	≈ 0	Absolute effect is estimated to be varying from > 0 to 19 more serious complications per 1000 patients in the ECTR group depending of the type of ECTR performed	⨁⨁⨁◯ MODERATE	CRITICAL

ECTR: Extracorporeal treatments, IHD: Intermittent hemodialysis, TPE: Therapeutic plasma exchange, CKRT: Continuous kidney replacement therapy, HP: Hemoperfusion, Pts = patients, PCC: Poison control center

“Requirement for extracorporeal life support,” “Length of requirement of vasopressors,” “Length of hospital stay,” “Length of ICU stay,” and “Sequelae” were outcomes ranked important or critical although no data were reported in the control group, so no comparison with the ECTR group could be performed

^a^Includes our systematic review of the literature on ECTR (37 patients from 32 case reports or case series) and 9 cohorts on standard care alone in β-adrenergic antagonists. No exclusion was based on the presence of co-ingestants or interventions

^b^Case reports published on effect of ECTR. Uncontrolled and unadjusted for confounders such as severity of poisoning, co-ingestions, supportive and standard care, and co-interventions. Confounding-by-indication is inevitable since ECTR was often attempted after other therapies had failed

^c^ECTR and standard care performed may not be generalizable to current practice (literature pre-dating 2000)

^d^Few events in small sample size, optimal information size criteria not met

^e^Publication bias is strongly suspected due to the study design (case reports published in toxicology either report very severe poisoning with/without impressive recovery with treatments attempted)

^f^Includes our systematic review of the literature on ECTR (9 case reports) and 4 cohorts on standard care alone in propranolol poisoning

^g^Includes our systematic review of the literature on ECTR (9 case reports) and 2 cohorts / case series on standard of care alone in sotalol poisoning

^h^Includes our systematic review of the literature on ECTR (9 case reports) and 2 cohorts on standard of care alone in atenolol poisoning

^i^Includes our systematic review of the literature on ECTR (3 case reports) and 1 case series on standard of care alone in sotalol poisoning

^j^For venous catheter insertion: serious complications include hemothorax, pneumothorax, hemomediastinum, hydromediastinum, hydrothorax, subcutaneous emphysema retroperitoneal hemorrhage, embolism, nerve injury, arteriovenous fistula, tamponade, and death. Hematoma and arterial puncture were judged not serious and thus excluded from this composite outcome. Deep venous thrombosis and infection complications were not included considering the short duration of catheter use

^k^Based 5 single-arm observational studies: 2 meta-analyses comparing serious mechanical complications associated with catheterization using or not an ultrasound, which included 6 RCTs in subclavian veins [322] and 11 in internal jugular veins [323]; 2 RCTs comparing major mechanical complications of different sites of catheterization [324, 325]; one large multicenter cohort study reporting all mechanical complications associated with catheterization [326]. Rare events were reported from case series and case reports

^l^Not rated down for inconsistency since heterogeneity was mainly explained by variation in site of insertion, use of ultrasound, experience of the operator, populations (adults and pediatric), urgency of catheter insertion, practice patterns, and methodological quality of studies

^m^Not rated down for indirectness since cannulation and catheter insertion was judged similar to the procedure for other indications

^n^Not rated down for imprecision since wide range reported explained by inconsistency

^o^The events in the control group are assumed to be zero (since no catheter is installed for ECTR); therefore, the magnitude of effect is at least expected to be large, which increases the confidence in the estimate of effect. Furthermore, none of the studies reported 95%CI which included the null value and all observed complications occurred in a very short timeframe (i.e., few hours)

^p^For IHD and CKRT: serious complications (air emboli, shock, and death) are exceedingly rare. Minor bleeding from heparin, transient hypotension, and electrolytes imbalance were judged not serious. For HP, serious complications include severe thrombocytopenia, major bleeding, and hemolysis. Transient hypotension, hypoglycemia, hypocalcemia, and thrombocytopenia were judged not serious. For TPE, serious complications include citrate toxicity, severe allergic reaction, arrhythmia, and vasovagal reaction. Hypotension, hypocalcemia, and urticaria were judged as not serious. All non-serious complications were excluded from this composite outcome

^q^IHD/CKRT: Based on 2 single-arm studies describing severe adverse events per 1000 treatments in large cohorts of patients [327, 328]. TPE: based on the 2 most recent one-arm studies reporting potential life-threatening adverse events [329, 330]. HP: Based on 2 small single-arm studies in poisoned patients [331, 332]. Rare events were reported in case series and case reports

^r^Assuming that patients in the control group would not receive any form of ECTR, the events in the control group would be zero; therefore, the magnitude of effect is at least expected to be large, which increases the confidence in the estimate of effect. Furthermore, none of the studies reported 95%CI which included the null value and all observed complications occurred in a very short timeframe (i.e., few hours)

## Discussion

### Recommendations

As per EXTRIP methods, the workgroup only voted on BAAs for which the number of patient clinical reports were sufficient. Although there were 4 reports for acebutolol and talinolol, they were not considered to be of sufficient quality to permit elaborations of recommendations.

#### General statements and indications for ECTR

**Propranolol**

- In patients severely poisoned with propranolol, we *recommend against* performing ECTR in addition to standard care rather than standard care alone (strong recommendation, very low quality evidence).

**Atenolol**

- In patients severely poisoned with atenolol and kidney impairment*, we suggest performing ECTR in addition to standard care rather than standard care alone when refractory bradycardia and hypotension is present (weak recommendation, very low quality evidence)
- In patients severely poisoned with atenolol and normal kidney function*, we make no recommendation* for or against performing ECTR in addition to standard care rather than standard care alone (no recommendation, very low quality evidence)

**Sotalol**

- In patients severely poisoned with sotalol and kidney impairment*, we suggest performing ECTR in addition to standard care rather than standard care alone when refractory bradycardia and hypotension and/or recurrent torsade de pointes is present (weak recommendation, very low quality of evidence)
- In patients severely poisoned with sotalol with normal kidney function*, we make no recommendation* for or against performing ECTR in addition to standard care rather than standard care alone (no recommendation, very low quality evidence).
- In patients severely poisoned with sotalol, *we suggest against* performing ECTR solely based on the QT interval (weak recommendation, very low quality evidence).

*“*Kidney impairment”* was defined as stage 3B, 4, or 5 CKD (i.e., eGFR < 45 mL/min/1.73m^2^) or AKI as KDIGO stage 2 or 3 AKI. In the absence of a baseline serum creatinine concentration, kidney impairment was defined as an eGFR < 45 mL/min/1.73m^2^ in adults; and in children with no baseline creatinine, the use of KDIGO criteria of AKI stage 2 and 3 after imputing a baseline serum creatinine using the Schwartz 2009 formula assuming 120 mL/min/1.73m^2^ of "normal" eGFR. The presence of oligo/anuria unresponsive to fluid resuscitation should be considered as impaired kidney function, regardless of serum creatinine concentration (See supplemental section)

#### Rationale

Severe BAA poisoning can lead to bradycardia and hypotension refractory to vasopressors and inotropes, occasionally causing death [57]. Assuming all other priority therapeutic measures are in place to mitigate BAA toxicity including involvement of a clinical toxicologist, the workgroup considered the use of ECTR for severe poisoning due to propranolol, atenolol, and sotalol.

Propranolol has a short half-life and a high endogenous clearance independent of kidney function. These attributes added to extensive protein binding make this drug non-dialyzable regardless of the ECTR used. Although the data were limited, ECTR did not appear to accelerate clinical recovery and the mortality from ECTR cases was higher than historical controls. For these reasons, the workgroup recommended against ECTR for propranolol poisoning (Median: 1.0/Upper quartile: 1.0/Disagreement index: 0.0).

Atenolol and sotalol both have endogenous clearances (and elimination half-lives) that are highly dependent on kidney function. The contribution of ECTR in patients with kidney impairment is considerable. The greater the impairment in kidney function, the greater the relative toxicokinetic effect of ECTR. Both are considered to be “*Dialyzable*” in patients with kidney impairment. Although the number of cases is small, clinical improvement from sotalol and atenolol poisoning appears to coincide with initiation of ECTR, especially when high efficiency techniques are used. It is conceivable that relevant patient-important outcomes (PIOs), such as length of vasopressor requirement, long-term sequelae, and mortality would be reduced with ECTR in this population. In patients who already have vascular access in place, the risk associated with insertion is already taken into account, so the risk-benefit ratio is even lower. The workgroup suggested ECTR in patients with impaired kidney function for both atenolol (Median: 7.0 / Lower quartile: 4.0 / Disagreement index: 0.59) and sotalol (Median: 7.0 / Lower quartile: 4.0 / Disagreement index: 0.59); the workgroup nevertheless acknowledged that the initiation of ECTR, even without net ultrafiltration, might exacerbate hemodynamic instability and may not be possible to perform. The benefit of ECTR is theoretically less for patients poisoned with atenolol or sotalol and normal kidney function, even if the addition of ECTR can approximately double total clearance; the duration of toxicity is expected to be much shorter in this population. For these reasons, the workgroup considered that, at the time of writing, the benefits and harms were balanced with considerable knowledge gaps and made no recommendation for patients poisoned with atenolol or sotalol and normal kidney function.

A major consideration for sotalol is its ability to cause QT prolongation, which is uncommon with other BAAs and can lead to life-endangering torsade de pointes, a poor prognostic indicator in sotalol poisoning. Obviously, the workgroup is not advocating ECTR for the treatment of torsade de pointes, as ECTR would not be technically feasible. However, recurrent torsade de pointes is indicative of severity and of a role for ECTR initiation. For non-recurrent torsades, ECTR is not justified. In the literature, there is no clear QTc duration cut-off which predicts torsade de pointes [134]. The risk of life-threatening cardiac events increases as the QTc gets longer than 500 ms [134, 135] and each 10-ms increase contributes to approximately a 5% to 7% exponential increase in risk. However, QT can be prolonged at therapeutic sotalol concentration. These findings support the recommendation of the workgroup not to perform ECTR solely based on QT prolongation.

Although monitoring of poison concentrations is useful in some settings, there remain too many uncertainties in the concentration-effect relationship to provide a threshold concentration for ECTR initiation in BAA poisoning. Hypotension and bradycardia are poorly related to atenolol concentrations [136], QT interval prolongation is correlated with sotalol concentrations but with considerable imprecision [45–48]. Further, only 7 out of 37 panelists had access to atenolol or sotalol assays and only 3 within 12 hours of it being ordered. Very few clinicians outside of large academic centers are likely to have access to BAA assays. The panel did recognize the value of a subtherapeutic concentration in excluding the need for ECTR. The panel emphasized that the indication for ECTR is likely to depend on the availability of ECLS, which should be instituted prior to ECTR assuming both are available in the same center, as it is simple to add a hemodialysis circuit to extracorporeal membrane oxygenation.

#### Research gaps

Additional pharmacokinetic data in ESKD patients are needed, especially during hemodialysis, for acebutolol (because of imprecision about sampling in studies), betaxolol, bopindolol, carteolol, cetamolol, nadolol, oxprenolol, pindolol, sotalol, and timolol. In addition, clinical cases of poisoning with toxicokinetic data of ECTR is required for acebutolol, atenolol, bisoprolol, metoprolol, nadolol and sotalol in patients with normal GFR or slightly impaired GFR.

Toxicokinetic/toxicodynamic relationships should better evaluate if serum concentrations can determine the utility of ECTR in clinical decision-making. Better prognostic markers on admission would also be useful to determine which subset of patients are most likely to benefit from ECTR.

The added value of ECTR to ECLS should be demonstrated. In patients with impaired kidney function, additional studies could help characterize if the transfer of an unstable patient for ECTR with or without ECLS could potentially be beneficial and within which timeframe this could be useful. If ECLS is unavailable in the initial center, studies could compare clinical outcomes associated with transfer for ECLS vs. hemodialysis alone at the initial center.

### Type of ECTR

In patients severely poisoned with atenolol or sotalol requiring ECTR: when all modalities are available, *we recommend using intermittent hemodialysis* rather than any other type of ECTR (strong recommendation, very low quality evidence).

#### Rationale

If ECTR is used for poison removal, then the most efficient modality at removing atenolol or sotalol should be selected, i.e., intermittent hemodialysis. In the rare circumstance that intermittent hemodialysis is unavailable but other techniques are, then hemoperfusion, CKRT, sustained low-efficiency dialysis (SLED), or prolonged intermittent renal replacement therapy (PIRRT) can be used, preferably the modality providing the best solute clearance and quickest to deliver. Although CKRT and other “slower” techniques such as SLED/PIRRT are often preferred for patients with hemodynamic compromise, this applies specifically to those requiring net ultrafiltration. It is therefore uncertain if CKRT or SLED/PIRRT would be better tolerated than intermittent hemodialysis in patients not requiring net ultrafiltration. It is acknowledged that all techniques may exacerbate hypotension to some extent for various causes including fluid and solute shifts, and electrolyte fluxes.

Regardless of technique, ECTR parameters should be optimized to enhance clearance (higher blood and effluent flows, filter/dialyzer with larger surface area) [137] and to reduce risk of hemodynamic compromise (priming of the ECTR circuit, lowering dialysate temperature, dialysate/replacement fluid without low potassium, calcium and magnesium concentrations, and minimizing net ultrafiltration).

Importantly, if dialysis is performed for sotalol poisoning, the input of a nephrologist is recommended to ensure that the serum magnesium concentration remains above 1 mmol/L and serum potassium concentration within 4.5-5 mmol/L to minimize the risk of dysrhythmias, including torsade de pointes. Magnesium may be added to the dialysate or administered intravenously to offset its elimination during ECTR.

#### Research gap

Data with hemoperfusion and high-cut off dialysis should be assessed in poisoning from highly protein-bound BAAs with reasonably low volume of distribution and plasma clearance such as penbutolol, oxprenolol, and carvedilol.

### Cessation of ECTR

In patients severely poisoned with atenolol or sotalol requiring ECTR, *we recommend stopping ECTR based on clinical improvement* (strong recommendation, very low quality of evidence)

#### Rationale

The indication to stop ECTR, once initiated, should be reliant on clinical indicators of improvement. These include appropriate heart rate and blood pressure for adequate end organ perfusion, weaning of ECLS, decreasing inotropic and vasopressor requirements, and sustained cessation of torsade de pointes if applicable. It is recognized that QT interval prolongation may persist even at therapeutic sotalol concentrations so the use of this target for cessation is not recommended. In addition, there is no predefined duration of ECTR to treat BAA poisoning as this will depend on the type and amount of BAA ingested, as well as the underlying kidney function in some cases. The workgroup suggested not to cease ECTR solely based on a target serum concentration, as safe thresholds are not well known, and assays are infrequently available to guide judgement.

Our work has several strengths. This is the first systematic review of the use of extracorporeal therapy in BAA poisoning. This systematic review summarizes the best evidence on the use of extracorporeal therapy in BAA poisoning using the most stringent guideline methodology (GRADE). No articles were rejected based on language or year of publication. It also provides clinical recommendations following a voting process using a two-round modified Delphi procedure from an international collaborative comprising recognized experts from various clinical specialties and resource settings. Limitations of the study are inherently associated with the quality of articles used for the drafting of recommendations. In many cases, details regarding these articles were of poor quality. There were insufficient data to draft recommendations on BAAs other than propranolol, atenolol, and sotalol due to the limited published evidence available; however, the workgroup acknowledged there was little clinical plausibility of a clinical benefit from ECTR for non-dialyzable BAAs such as betaxolol, carvedilol, esmolol, labetalol, mepindolol, and timolol.

## Conclusion

In conclusion, poisoning from BAAs can cause serious toxicity and death. β-adrenergic antagonists have different physicochemical properties and pharmacokinetics which will affect their removal by ECTR. The EXTRIP workgroup assessed propranolol as non-dialyzable. Atenolol as well as sotalol were assessed as dialyzable in patients with kidney impairment and the workgroup suggests ECTR in patients severely poisoned with these drugs when aforementioned indications are present.

## Supplementary Information

**Additional file 1**. Detailed methods and glossary.

## Data Availability

The data underlying this article will be shared on reasonable request to the corresponding author.

## Abbreviations

BAA: Beta-adrenergic antagonists
CKD: Chronic kidney disease
CKRT: Continuous kidney replacement therapy
CL: Clearance
ECLS: Extracorporeal life support
ECTR: Extracorporeal treatments
ESKD: End-stage kidney disease
EXTRIP: The EXtracorporeal TReatments In Poisoning workgroup
F: Bioavailability
GFR: Glomerular filtration rate
HD: Hemodialysis
HF: Hemofiltration
HP: Hemoperfusion
ICU: Intensive care unit
IHD: Intermittent hemodialysis
IQR: Interquartile range
Met: Metabolite
MW: Molecular weight
N/A: Not available
PCC: Poison control center
PD: Peritoneal dialysis
PIO: Patient-important outcomes
PK: Pharmacokinetics
Pts: Patients
T_1/2_: Elimination half-life
TK: Toxicokinetics
T_MAX_: Time to maximum concentration
TPE: Therapeutic plasma exchange
V_D_: Volume of distribution
