# Supplementary material for: Extracorporeal treatment for poisoning to beta-adrenergic antagonists: systematic review and recommendations from the EXTRIP workgroup
Source: Crit Care. 2021 Jun 10;25:201. doi: 10.1186/s13054-021-03585-7 (PMC8194226; doi:10.1186/s13054-021-03585-7)
Supplement: Supplementary file 1 — Additional file 1. Detailed methods and glossary. [file 13054_2021_3585_MOESM1_ESM.pdf]

Additional file 1: methods, glossary and references

Table of contents

A) METHODS ………………………………………………………………………page 2
B) GLOSSARY……………………………………………………………………...page 11
C) REFERENCES…………………………………………………………………..page 12

EXTRIP clinical practice guideline

As defined by the Institute of Medicine in 2011, clinical practice guidelines are statements that include recommendations intended to optimize patient care by assisting practitioners and patients in making decisions about appropriate health care for specific clinical circumstances.[1] They are informed by a systematic review of evidence and an assessment of the benefits and harms of alternative care options. Attributes of good guidelines include validity, reliability, reproducibility, clinical applicability, clinical flexibility, clarity, multidisciplinary process, review of evidence, and documentation.

The EXTRIP-2 workgroup pursued a second phase of the international multidisciplinary effort started in 2010[2]. Using established methodology[3], the EXTRIP workgroup reviewed the literature and developed recommendations on the use of extracorporeal treatments (*ECTR, see glossary*) in the context of poisoning (*see glossary*) for a new set of 10 poisons (*see glossary*). More specifically, the effect of ECTRs in addition to standard care was measured against standard care alone. Potential benefits of ECTRs were balanced with potential harms from the procedure. Outcomes measured included mortality, relevant clinical and physiological end points, complications associated with ECTR procedure, as well as the extent of extracorporeal removal of the poison (i.e., dialyzability, see glossary see below). When applicable, different populations (including end-stage kidney disease and pediatric patients), types of poisoning (acute, acute-on-chronic, and chronic), and types of ECTR were evaluated. When developing recommendations, variation in clinical practice in different settings across the globe was considered especially regarding resource use, costs, and availability of antidotes and ECTR.[4] Implementation issues were addressed when appropriate.

Workgroup composition

The EXTRIP workgroup is an international collaborative comprising recognized experts from various clinical specialties (medical toxicology, emergency medicine, nephrology, critical care, pediatrics, and pharmacology). The EXTRIP-2 workgroup renewed the participation of 15 members and invited 22 new experts to join the workgroup and increase representativeness across the world (Table S1). Six co-chairs (M.G., S.G., R.S.H., V.L., T.D.N., and D.M.R.), of which, one is a non-voting guideline methodologist (V.L.) led and supervised the different aspects of the guideline development.

Table S1. Represented societies

African Federation of Emergency Medicine	European Association of Poison Centres and Clinical Toxicologists
Acute Dialysis Quality initiative	European Renal Association-European Dialysis and Transplant Association
American College of Clinical Pharmacology	European Society for Emergency Medicine
American Academy of Clinical Toxicology	European Society of Intensive Care Medicine
American College of Emergency Physicians	Faculty of Intensive Care Medicine
American College of Medical Toxicology	International Pediatric Nephrology Association
American Society of Nephrology	International Society of Nephrology
American Society of Pediatric Nephrology	Middle East and North Africa Clinical Toxicology Association
Asia Pacific Association of Medical Toxicology*	National Kidney Foundation
Asian Pacific Nephrology Association	Pediatric Continuous Renal Replacement Therapy
Australian and New Zealand Intensive Care Society*	Pediatric Critical Care Medicine
Australian and New Zealand Society of Nephrology*	Society of Academic Emergency Medicine
Brazilian Association of Information Centres and Toxicologic Assistance*	Society of Critical Care Medicine
Brazilian Society of Toxicology	The Renal Association
Canadian Association of Emergency Physicians	
Chinese College of Emergency Physicians	

Chinese Medical Association	

*This representation does not signify endorsement. Recommendations will be submitted to societies for review and potential endorsement.

Disclosure and management of potential COI

All prospective members were required to disclose any actual, potential, or perceived COI prior to inclusion in the workgroup. The disclosures were used to categorize the members as cleared for full participation, allowed to participate with recusal from certain aspects of guideline development, or disqualified from participation. The co-chairs remained free of any financial COI during the entire guideline development process, meaning avoidance of interests and relationships with pharmaceutical or device companies pertaining to the topic of poisoning. Members were required to disclose to the co-chairs any new activities that had the potential to be viewed as a COI prior to engaging in the activity, at the beginning of face-to-face meeting, and before submission of the manuscript. Co-chairs determined if specific activities were allowed under the COI rules. All COIs deemed as potential appearance of a conflict of interest were required to be included in the manuscript.

Framing of the clinical question

An initial list of relevant clinical questions was developed for each poison, reviewed by the co-chairs, and then approved by the entire workgroup prior to the first iteration of EXTRIP-2. Clinical questions were formulated following the standard PICO format. Patient population and specific subpopulations (e.g., type of poisoning / exposure, patients with/without kidney impairment) as well as interventions of interest (e.g., type of ECTR) were defined for all clinical questions, as applicable. Comparator(s) of interest were explicitly defined as the standard care (with/without antidotes) for every poison (Table S2).

Table S2: Standard care for each poison reviewed

Poison / drug	"Standard care"	Targeted comparator	Comparison
Ethylene glycol	Standard 1: ABC, decontamination, bicarbonate, pyridoxine/thiamine, ethanol	None	Standard1 vs Standard1 + ECTR
	Standard 2: ABC, decontamination, bicarbonate, pyridoxine/thiamine, fomepizole		Standard2 vs Standard2 + ECTR
Methotrexate	ABC, decontamination, urine alkalinisation, folinic acid, blood products<br>Considerations for decontamination	Glucarpidase	Standard care vs<br>Standard care + glucarpidase vs<br>Standard care + ECTR vs<br>Standard care + glucarpidase + ECTR
Dabigatran	ABC, decontamination, FFP, prothrombin complex<br>Considerations for FVIII / FEIBA, rhVIIa	Idarucizumab	Standard care vs<br>Standard care + idarucizumab vs<br>Standard care + ECTR vs<br>Standard care + idarucizumab + ECTR
Isoniazid	ABC, decontamination, pyridoxine, benzodiazepines	None	Standard care vs standard care + ECTR
Beta-adrenergic antagonists	ABC (including cardiac pacing), decontamination, vasopressors, inotropes, high-dose insulin euglycemia therapy, atropine, glucagon<br>Considerations for ECLS	None	Standard care vs standard care + ECTR
Calcium-channel blockers	ABC (including pacing), decontamination, vasopressors, inotropes, IV calcium, high-dose insulin euglycemia therapy<br>Considerations for ECLS	None	Standard care vs standard care + ECTR
Quinine / chloroquine / hydroxychloroquine	ABC (including pacing), decontamination, MDAC, benzodiazepines<br>Considerations for ECLS	None	Standard care vs standard care + ECTR
Baclofen	ABC, decontamination, benzodiazepines	None	Standard care vs standard care + ECTR
Gabapentin / pregabalin	ABC, decontamination	None	Standard care vs standard care + ECTR

Amanita / amanitin / amatoxin	ABC, decontamination, NAC, MDAC<br>Considerations for Silibinin / silymarin, biliary drainage, rifampicin	None	Standard care vs standard care + ECTR

NAC: N-acetylcysteine, MDAC: multidose activated charcoal, ECLS: Extracorporeal life support, ECTR: Extracorporeal treatment, FFP: fresh frozen plasma

Selection and ranking of patient-important outcomes

All outcomes of interest were identified *a priori,* and the workgroup members explicitly rated their relative importance for decision making. In general, patient-important outcomes considered were: 30-day mortality, serious or permanent end-organ injury specific to each poison reviewed, serious complications of catheter insertion (see glossary), serious complications of ECTR (see glossary), length of ICU stay, length of hospital stay, and costs. Ranking of the outcomes by importance for decision-making (either “critical”, ‘important but not critical” or “of limited importance”) was determined by consensus for each poison reviewed and for PICO question developed.

Dialyzability was reported as a separate outcome: the workgroup acknowledged that excellent poison removal does not correlate with clinical improvement for certain poisons. Conversely, clinical improvement can sometimes be observed despite poor extracorporeal removability.

Systematic review: balance of benefits and harms of ECTR in poisoning to beta-adrenergic antagonists

The objectives of this systematic review of the literature were to: 1) summarize the balance of benefits and harms of ECTR on patient important outcomes, and 2) describe the toxicokinetic outcomes of ECTR in the context of severe poisoning to beta-adrenergic antagonists.

One health sciences librarian (E.G.) in collaboration with one co-chair (M.G) and the methodologist (V.L.) designed literature searches to address clinical questions. PubMed / MEDLINE, and EMBASE were searched initially for prior systematic reviews that have addressed similar topics. Based on the result of this initial landscape assessment of the literature and based on *a priori* knowledge of the literature, an assumption was made by the workgroup that no or very few studies directly comparing the effect of ECTR to standard care would be identified. Thus, the initial search strategy was designed to systematically search and identify all studies describing the effect of ECTR on patient important outcomes or toxicokinetic outcomes. If the initial search of the literature did not identify comparative studies in which ECTR was measured against standard care alone (with or without an antidote, if applicable), complementary searches was designed to systematically search and identify studies describing patient important outcomes and prognostic factors in patients receiving the standard care alone. In addition to the mentioned systematic reviews, a complementary search was designed to identify the best available evidence describing harms associated with catheter insertion and ECTR procedure. The systematic review protocol is reported in accordance with the PRISMA-P 2015 Checklist (Table S3), as appropriate.

Table S3: PRISMA-P 2015 checklist [5]

Section and topic			#	Checklist item	Information reported
ADMINISTRATIVE INFORMATION	Title	Identification	1a	Identify the report as a protocol of a systematic review	Y
		Update	1b	If the protocol is for an update of a previous systematic review, identify as such	N/A
	Registration		2	If registered, provide the name of the registry (e.g., PROSPERO) and registration number	N
	Authors	Contact	3a	Provide name, institutional affiliation, and e-mail address of all protocol authors; provide physical mailing address of corresponding author	Y
		Contributions	3b	Describe contributions of protocol authors and identify the guarantor of the review	Y
	Amendments		4	If the protocol represents an amendment of a previously completed or published protocol, identify as such and list changes; otherwise, state plan for documenting important protocol amendments	N/A

	**Support**	Sources	5a	Indicate sources of financial or other support for the review	**Y**
		Sponsor	5b	Provide name for the review funder and/or sponsor	**Y**
		Role of sponsor or funder	5c	Describe roles of funder(s), sponsor(s), and/or institution(s), if any, in developing the protocol	**Y**
**INTRODUCTION**	**Rationale**		6	Describe the rationale for the review in the context of what is already known	**Y**
	**Objectives**		7	Provide an explicit statement of the question(s) the review will address with reference to participants, interventions, comparators, and outcomes (PICO)	**Y**
**METHODS**	**Eligibility criteria**		8	Specify the study characteristics (such as PICO, study design, setting, time frame) and report characteristics (such as years considered, language, publication status) to be used as criteria for eligibility for the review	**Y**
	**Information sources**		9	Describe all intended information sources (such as electronic databases, contact with study authors, trial registers or other grey literature sources) with planned dates of coverage	**Y**
	**Search strategy**		10	Present draft of search strategy to be used for at least one electronic database, including planned limits, such that it could be repeated	**Y**
	**Study records**	Data management	11a	Describe the mechanism(s) that will be used to manage records and data throughout the review	**Y**
		Selection process	11b	State the process that will be used for selecting studies (such as two independent reviewers) through each phase of the review (that is, screening, eligibility, and inclusion in meta-analysis)	**Y**
		Data collection process	11c	Describe planned method of extracting data from reports (e.g., piloting forms, done independently, in duplicate), any processes for obtaining and confirming data from investigators	**Y**
	**Data items**		12	List and define all variables for which data will be sought (such as PICO items, funding sources), any pre-planned data assumptions and simplifications	**Y**
	**Outcomes and prioritization**		13	List and define all outcomes for which data will be sought, including prioritization of main and additional outcomes, with rationale	**Y**
	**Risk of bias in individual studies**		14	Describe anticipated methods for assessing risk of bias of individual studies, including whether this will be done at the outcome or study level, or both; state how this information will be used in data synthesis	**Y**
	**Synthesis**		15a	Describe criteria under which study data will be quantitatively synthesized	**Y**
			15b	If data are appropriate for quantitative synthesis, describe planned summary measures, methods of handling data, and methods of combining data from studies, including any planned exploration of consistency (such as I^2, Kendall's tau)	**N/A**
			15c	Describe any proposed additional analyses (e.g., sensitivity or subgroup analyses, meta-regression)	**N/A**
			15d	If quantitative synthesis is not appropriate, describe the type of summary planned	**N/A**
	**Meta-bias(es)**		16	Specify any planned assessment of meta-bias(es) (such as publication bias across studies, selective reporting within studies)	**N/A**
	**Confidence in cumulative evidence**		17	Describe how the strength of the body of evidence will be assessed (such as GRADE)	**Y**

Legend: Y; yes, N; no, N/A; not applicable

Literature search: Effect of ECTR

Eligibility criteria: as per EXTRIP initial methodology (reference), eligibility criteria for study selection were based on:

-Types of study design: all types, including randomized controlled trials, non-randomized studies, one-arm studies, case series/case reports, abstracts from scientific and clinical meetings. Modeling studies, animal experiments and *in vitro* experiments are accepted if the methods and results are interpretable and correlated in humans.

-Type of participants: all patients with severe poisoning to beta-adrenergic antagonists (*see glossary*). There is no restriction on the context of the poisoning (acute, chronic, acute-on-chronic) or heterogeneity of the participants (e.g., special populations such as pediatric, chronic kidney disease, pregnancy).

-Types of interventions: all types of ECTR if instituted at least partially for the purpose of poison removal. Studies in which ECTR was instituted exclusively for other indications such as kidney replacement therapy for acute kidney injury or albumin dialysis for hepatic failure will be excluded.

-Types of comparators: standard care without ECTR (pre-defined, see Table above)

-Types of outcomes: patient-important outcomes ranked critical or important for decision-making were: mortality (critical), duration of QT prolongation for sotalol (important), serious complications of catheter insertion (critical), serious complications of ECTR (critical), length of ICU stay (important), requirement for extracorporeal life support (important), length of requirement of vasopressors (important), sequelae (critical), and length of hospital stay (important). Dialyzability was reported as a separate outcome.

Search strategy: a search strategy was first developed in Pubmed/MEDLINE and then translated into the other databases, as appropriate. The following electronic databases were searched: Medline/PubMed, EMBASE, Cochrane Library (Review and Central), Conference proceedings/meeting abstracts of the EAPCCT and NACCT annual meetings (since 2002). All databases were searched from the date of inception to March 1st, 2019. Bibliographies of all full-text articles were manually searched for additional papers. There were no language exclusion criteria nor any other publication restrictions.

Literature search: Standard care

As mentioned above, if the initial search of the literature did not identify comparative studies in which ECTR was measured against standard care alone (with or without an antidote, if applicable), a specific search for the control group was developed.

Eligibility criteria for study selection were based on:

-Types of study design: one-arm studies, cohorts, case series, case reports (if the former designs were not identified)

-Types of participants: all patients with severe poisoning to beta-adrenergic antagonists (pre-defined). There is no restriction on the context of the poisoning (acute, acute-on-chronic or chronic) or heterogeneity of the participants (e.g., special populations such as pediatric, chronic kidney disease, hepatic insufficiency, pregnancy).

-Types of outcomes: patient-important outcomes (as defined above)

Search strategy: a search strategy was first developed in Pubmed/MEDLINE and then translated into the other databases, as appropriate. The following electronic databases were searched: Medline/PubMed, EMBASE, Cochrane Library (Review and Central), Conference proceedings/meeting abstracts of the EAPCCT and NACCT annual meetings (since 2002). All databases were searched from the date of inception to March 1st, 2019. Bibliographies of all full-text articles were manually searched for additional papers. There were no language exclusion criteria nor any other publication restrictions.

The search strategy used was as follows: ((dialysis or hemodialysis or haemodialysis or hemoperfusion or haemoperfusion or plasmapheresis or plasmaphaeresis or hemofiltration or haemofiltration or hemodiafiltration or haemodiafiltration or plasma exchange or CRRT or CVV* or CKRT or exchange transfusion) and (beta blocke* or beta adrenergic or acebutolol or alprenolol or atenolol or betaxolol or bisoprolol or bopindol or carteolol or carvedilol or celiprolol or cetamolol or esmolol or labetalol or medroxalol or mepindol or metipranolol or metoprolol or nadolol or nebivolol or oxprenolol or penbutolol or pindolol or practolol or prenalterol or propranolol or sotalol or talindolol or talinolol or timolol or tolamolol)).

Literature search: ECTR harms

Potential complications of ECTRs, either from catheterization or from the procedure itself, were comprehensively searched in PubMed/Medline for the best available evidence (randomized controlled trials and large cohort studies). Non-poisoning data were accepted, although adapted to acute and short-term scenarios (e.g. long-term infectious risks of catheters were not considered). The keyword search strategy was: ((kidney or renal) replacement therapy) or dialysis or h?moperfusion or h?mofiltration* or h?modialysis or h?modialfiltration or h?moadsorption or plasmaph?resis or (plasma exchange) or mars or prometheus or (liver support device*) or C?RT or C?VH* or (exchange transfusion)) AND (complication OR safety OR side effect OR adverse event OR harm OR adverse effect)

Update of literature searches

This search strategy was reconducted prior to submission on October 23rd, 2020, and pertinent articles not initially identified were submitted to the workgroup to assess if they modified the recommendations.

Screening of articles

All citations were entered into Endnote (version X9, Clarivate Analytics Inc., Philadelphia, PA). Two experienced workgroup members (J.B. and M.G.) screened the study titles and abstracts independently in duplicate to

determine eligibility for full-text assessment. Any citation that was considered of interest was included for the full-text assessment. Prior to the full-text screening, all studies published in non-English languages were translated in English by designated translators. Subsequently, the same members examined the full texts to assess inclusion and exclusion criteria. Disagreements was resolved by consensus or by involvement of the methodologist (V.L.).

Data management and extraction

Relevant data was extracted from Endnote and abstracted into a standardized data extraction tool using Microsoft Excel software (Version 2102). One chair (M.G.) was responsible for the master copy. Two experienced workgroup members (J.B. and M.G.) extracted the data into Microsoft Excel software (version 2102); Both a third member (G.S.) and the methodologist (V.L.) reviewed both versions, resolved inconsistencies and consolidated the data in one master flowsheet.

The following data for each eligible study were extracted into the standardized data extraction form:

-Study characteristics: first author, publication year, study design, number of treatment arms
-Baseline patient characteristics: age, gender, population size, kidney function, comorbidities
-Exposure: route of exposure, dose, length of exposure, co-ingestion, intent, time from ingestion to admission
-Clinical characteristics of poisoning: Hypotension, dysrhythmia, bradycardia, respiratory depression, mental status alteration, seizures, QT interval duration, QRS complex duration, acid-base abnormalities, creatinine, lactate, potassium, glucose, and blood sampling of poison / metabolites (on admission, peak value)
-Non-ECTR management: vasopressors, inotropes, intravenous, bicarbonate, mechanical ventilation, decontamination, atropine, glucagon, calcium, high-dose insulin euglycemic therapy, extracorporeal life support, lipid emulsion
-ECTR: type, indication, interval between admission and ECTR, number of sessions, duration of session, catheter, prescription (e.g., anticoagulation, dialyzer, dialysate)
-Toxicokinetics (protein binding, volume of distribution, endogenous clearance, half-life (pre, per, and post-ECTR), extraction ratio, ECTR clearance, removal by ECTR, removal in urine, presence and magnitude of rebound. These were calculated by the data extractors if not reported.
-Clinical outcome: death, length of hospital stay, reported improvement during ECTR, length of ICU stay, sequelae, complications of ECTR, duration of QT prolongation due to sotalol, requirement for extracorporeal life support, length of requirement of vasopressors, sequelae. Dialyzability was reported as a separate outcome.

Dialyzability

Dialyzability was defined a priori as the ability of any ECTR to remove a clinically significant percentage of the total body burden of the poison.[3] Different criteria were used to semi-quantitively categorize the dialyzability of the poison for each ECTR (Table S4).

Table S4. EXTRIP criteria for assessing dialyzability

**Dialyzability†**	**Primary criterion**	**Alternative criterion 1**	**Alternative criterion 2**	**Alternative criterion 3**
	**% Removed‡**	**CL_{ECTR} / CL_{TOT} (%)§**	**$T_{1/2\ ECTR}$ / $T_{1/2}$ (%)¶**	**RE_{ECTR} / RE_{TOT} (%)#**
**D**, Dialyzable	>30%	>75%	<25%	>75%
**M**, Moderately dialyzable	>10-30%	>50-75%	>25-50%	>50-75%
**S**, Slightly dialyzable	≥3-10%	≥25-50%	≥50-75%	≥25-50%
**N**, Not dialyzable	<3%	<25%	<75%	<25%

† Applicable to all modalities of ECTR, including hemodialysis, hemoperfusion, hemofiltration.
‡ Corresponds to % removal of ingested dose (adjusted for bioavailability) or total body burden, adjusted for a 6-hour ECTR period.
§ Corresponds to the clearance by ECTR (CL_{ECTR}) compared to the total clearance (CL_{TOT} i.e., endogenous + ECTR clearance)

¶ Corresponds to the apparent half-life during ECTR ($T_{1/2\ ECTR}$) compared to the apparent half-life off ECTR ($T_{1/2}$).
# Corresponds to the amount removed by ECTR (RE_{ECTR}) compared to the amount eliminated from the body (RE_{TOT}) measured during the same period.
These criteria should only be applied if measured or calculated (<u>not reported</u>) endogenous $T_{1/2}$ is > 4h (otherwise, the benefit of ECTR is not likely to be considered clinically relevant). Furthermore, the primary criterion is preferred for poisons having a large V_D (>1-2L/Kg)

If toxicokinetic data was insufficient, pharmacokinetic publications of ECTR removal of therapeutic drugs in non-poisoning situations were used, with the understanding that there may be inherent differences between both scenarios (e.g., alterations in protein binding, endogenous clearance, bioavailability, etc…). Despite these differences, these studies are often better designed (i.e., prospective, more patients) and can better explain the dialyzability of a poison.

Quality assessment of individual studies

The quality of reporting and risk for bias of each included study was assessed independently by two workgroup members (J.B. and M.G). Disagreements was resolved by consensus or involvement of the methodologist (V.L.). Risk of bias was assessed using the Cochrane Risk of Bias Tool for RCTs and the Risk of Bias Instrument for Non-randomized Studies – of Interventions (ROBINS-I).[6, 7]

The quality of individual studies reporting on toxicokinetic outcomes was assessed according to a pre-defined set of criteria (Table S5) and then summarized into a quality of the overall evidence (Table S6). If the latter was judged low or very low, literature from non-poisoning contexts was also considered, such as CKD pharmacokinetics, animal, and *in vitro* studies.

Table S5: Quality of individual studies for toxicokinetic outcomes.

**Quality of individual studies**	**Interpretation and application to individual studies**
High	Sufficient TK/PK data present; % removed is reported or can be calculated; reported calculations are appropriate.
Moderate	Sufficient TK/PK data present, but % removed is NOT reported or CANNOT be calculated; reported calculations (e.g., CL_{EC}/CL_{TOT}) are appropriate.
Low	Sufficient PK parameters may be reported, but supporting data absent or suspect, reported calculations inappropriate, or other serious limitations exist.
Very Low	Sufficient PK parameters and supporting data not adequately reported, questionable or no calculations reported. However, based on theoretical knowledge of V_D, protein binding, CL_{SYS}, molecular weight, etc., some assumptions can be made about dialyzability.
Reject	Questionable parameters reported with no supporting data, fatal flaw in study design.

Table S6. Quality of evidence for toxicokinetic outcomes

**Quality of evidence**	**Reporting**	**Interpretation**
High	A	We are confident that the true effect lies close to our estimate of the effect.
Moderate	B	The true effect is likely to be close to our estimate of the effect, but there is a possibility that it is substantially different.
Low*	C	The true effect may be substantially different from our estimate of the effect.
Very Low*	D	Our estimate of the effect is just a guess, and it is very likely that the true effect is substantially different from our estimate of the effect*.

**If the quality of the evidence is low or very low, literature from non-poisoning contexts may be used, such as pharmacokinetic studies in ESKD populations, animal, and in-vitro studies.*

Data synthesis

For dichotomous outcome measures, pooled estimate of the proportion of patients across studies with the corresponding 95% confidence interval (CI). was calculated. For continuous outcomes, a pooled estimate of the mean and a corresponding 95% CI or the median with first and third quartiles, was calculated, as appropriate. Due to the presumed lack of comparative studies on the effect of ECTR vs standard care alone, no meta-analysis was planned *a priori*.

Summary of evidence and quality of evidence

Evidence summaries for each question were prepared by the workgroup members assigned to a specific drug or poison in collaboration with the methodologist. For a clinical question to be formally developed into a recommendation, the workgroup agreed *a priori* that a minimum of 3 reported cases describing clinical outcomes for a specific poison was required.

In absence of direct comparison between the intervention (standard care plus ECTR) and comparator (standard care alone), the members selected the publications reporting controls that most closely resembled patients from the ECTR group (especially with regards to severity of poisoning) For example, controls admitted to the ICU were considered more likely to be comparable to the ECTR cohort than patients reported to poison control centers.

The quality of the evidence (Figure S1) was initially assessed for each critical and important outcome, and then for each recommendation using the Grading of Recommendations Assessment, Development and Evaluation (GRADE) approach.[8, 9] The quality of evidence will be assessed across the domains of risk of bias, consistency, directness, precision and publication bias. Additional domains may be considered where appropriate. Quality will be adjudicated as high (further research is very unlikely to change our confidence in the estimate of effect), moderate (further research is likely to have an important impact on our confidence in the estimate of effect and may change the estimate), low (further research is very likely to have an important impact on our confidence in the estimate of effect and is likely to change the estimate), or very low (very uncertain about the estimate of effect). GRADE evidence profile tables were developed in GRADEpro Guideline Development Tool (GRADEpro GDT: GRADEpro Guideline Development Tool [Software]. McMaster University, 2015 (developed by Evidence Prime, Inc.). Available from gradepro.org). The summaries of evidence were reviewed by all workgroup members prior to drafting recommendations.

Figure S1: Approach to and implications of rating the quality of the evidence and strength of recommendations using the GRADE methodology.

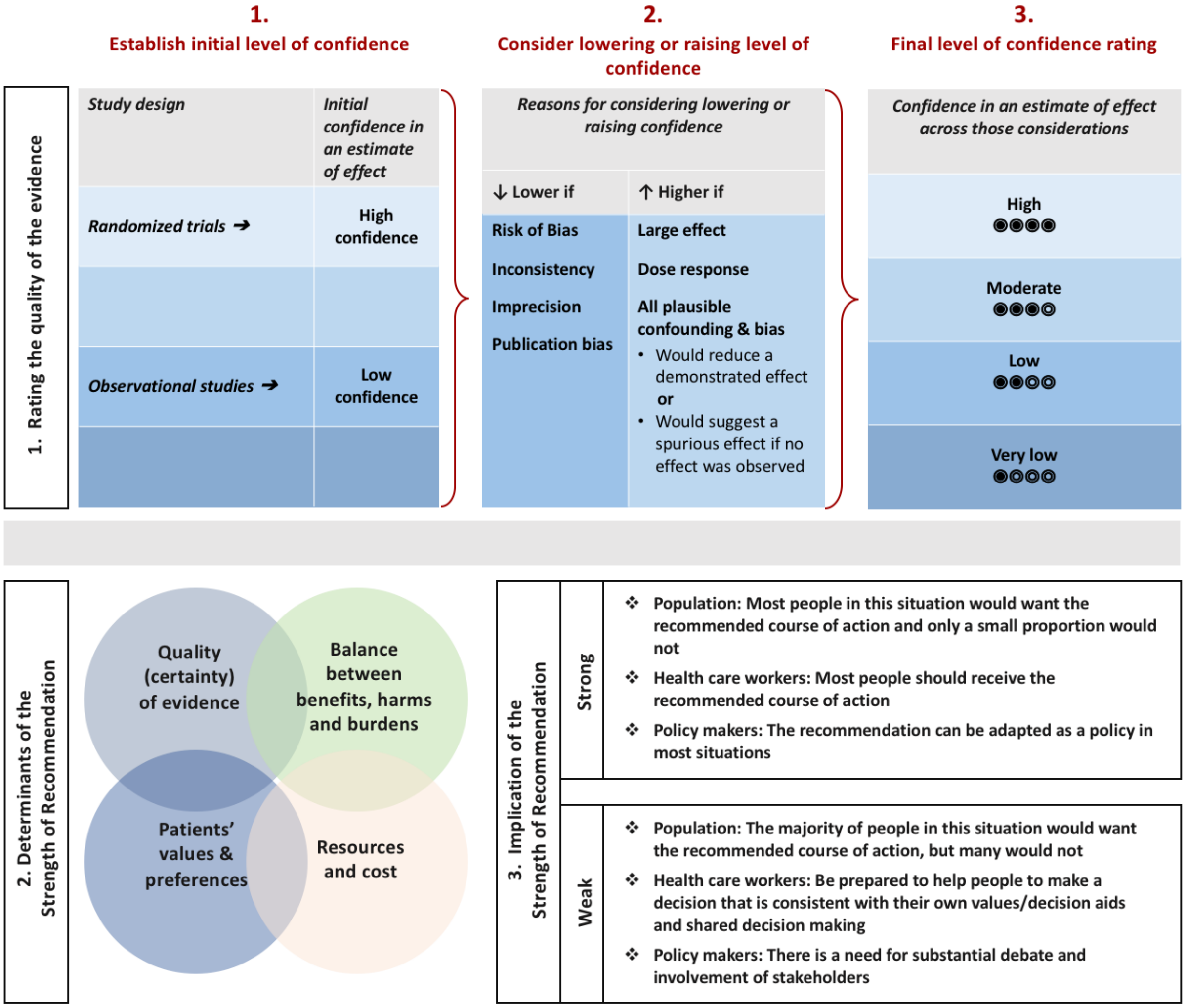

Unrestricted use of the figure granted by the GRADE working group.

Development of clinical recommendations

The workgroup considered core elements of the GRADE evidence in the decision process, including the quality of evidence and balance between desirable and undesirable effects. Additional domains were acknowledged where applicable (feasibility, resource use, acceptability). A web-based international survey performed by the EXTRIP workgroup provided the main source of evidence to address other important considerations such as resource use, costs, and availability of ECTR worldwide[2]. This survey also informed potential implementation issues. EXTRIP discussed organizational barriers in health care centers that may not have the resources and technical expertise to adopt the recommendations. Recommendations will also address situations where ECTR was previously performed but no longer supported.

For all recommendations, the workgroup members voted to reach agreement for final recommendations. The voting process followed the same set of rules as for EXTRIP-1 (i.e., anonymous online voting consisting of two-round modified Delphi with each statement voted on a 9-point Likert scale and final results interpreted according to EXTRIP voting rules based on median, lower or upper quartile (LQ or UQ) as appropriate, and disagreement index (DI) as calculated using RAND/UCLA Appropriateness Method (Figure S2)) and was performed using SimpleSurvey software.

Figure S2: Voting process for recommendations.

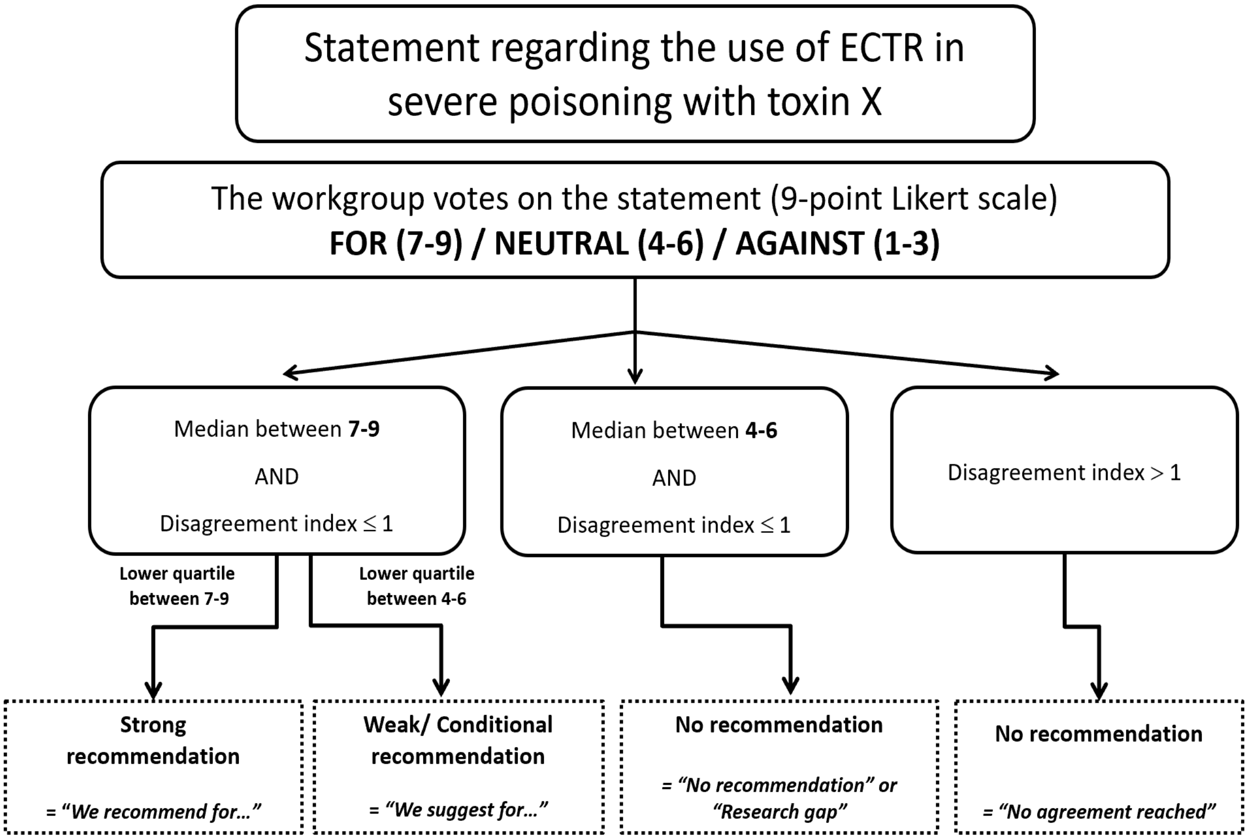

These recommendations can be formulated for or against a course of action (the figure only shows voting results FOR a course of action)

All recommendations were labelled as either "strong" or "weak/conditional" according to the GRADE approach. The words "we recommend" indicate strong recommendations and "we suggest" indicate weak/ conditional recommendations. Figure S1 provides the suggested interpretation of strong and weak/conditional recommendations for patients, clinicians, and health care policy makers. In summary, a "strong" recommendation implies that most individuals in this situation would want the recommended course of action and only a small proportion would not, while a "weak/conditional" recommendation means that the majority of individuals in this situation would want the suggested course of action, but many would not. The latter recognizes that different choices will be appropriate for different patients and that clinicians must help each patient to arrive at a management decision consistent with their own values and preferences.

High-quality evidence was expected to be lacking for the majority of recommendations. According to GRADE guidance, strong recommendations in the setting of lower-quality evidence were only assigned when the workgroup members believed they conformed to one or several paradigmatic conditions. As per GRADE guidance on discordant recommendations[6], two paradigmatic situations presented in the development of EXTRIP-2 guideline: 1) low-quality evidence suggested benefit in a life-threatening situation (with evidence regarding harms being low or high), and 2) when low-quality evidence suggested benefit and high-quality evidence suggested harm.

If the workgroup could not make a recommendation for or against a particular management strategy due to either 1) a close balance between the benefits and harms (no recommendation), or 2) insufficient evidence making a recommendation too speculative (research gap), either a "no recommendation" or "research gap" recommendation was formulated (a "neutral recommendation" interpreted as a "reasonable course of action" was no longer accepted). Although there is arguably ongoing need for research on virtually all of the topics considered in this guideline, "Research Needs" were noted for recommendations in which the need was believed by the workgroup to be particularly relevant.

The entire workgroup gathered in Montreal, Canada in November 2019 for the presentation of evidence summaries and the development of the recommendations for the ten new poisons. The subgroup assigned to a specific poison participated in the preparation of the draft guideline in collaboration with co-chairs.

Updating process

Ongoing screening of the literature will take place to determine the need for revisions based on the likelihood that any new data will have an impact on the recommendations. If necessary, the entire workgroup will reconvene to discuss potential changes.

Glossary

-*Acute liver failure*: (a) the presence of hepatic encephalopathy of any degree; (b) evidence of moderately severe coagulopathy [i.e., international normalized ratio (INR) ≥1.5]; (c) presumed onset of acute illness of <26 weeks; and (d) the absence of cirrhosis
-*Acute liver injury*: (a) evidence of moderately severe coagulopathy (INR ≥2.0); (b) presumed onset of acute illness <26 weeks; and (c) the absence of cirrhosis
-*Altered mental status*: poison-induced impairment in at least one brain function (cognition, alertness, or orientation), in the absence of another cause
-*Clearance*: The volume of blood (or solvent) cleared of poison per unit time, typically reported in units of mL/min. Importantly, CL_{ECTR} represents solute clearance due exclusively to ECTR and is independent of endogenous clearance (CL_{ENDO}; the sum of underlying renal and non-renal clearances). CL_{TOT} refers to total clearance and is the sum of CL_{ECTR} and CL_{ENDO}
-*Coma*: a deep state of unconsciousness as defined per the American Academy of Neurology
-*Dialyzability*: This term reflects the ability of ECTR to remove a clinically significant percentage of the total body burden of the poison
-*Extracorporeal treatment (ECTR)*: A treatment, occurring outside the body, which promotes poison removal by mechanisms different from endogenous pathways. ECTR includes HD, continuous renal replacement therapy, extended dialysis, peritoneal dialysis (although technically occurring in the body), hemofiltration, hemodiafiltration, hemoperfusion, therapeutic plasma exchange and albumin/ "liver" dialysis
-*Kidney impairment*: CKD as stage 3B, 4, or 5 CKD (i.e., eGFR < 45 mL/min/1.73m^2) or AKI as KDIGO stage 2 or 3 AKI. In the absence of a baseline creatinine, a GFR < 45 mL/min in adults; in children with no baseline creatinine, the use of KDIGO criteria of AKI stage 2 and 3 after imputing a baseline serum creatinine using the Schwartz 2009 formula assuming 120 mL/min of "normal" eGFR. The presence of oligo/anuria unresponsive to fluid resuscitation should be considered as impaired kidney function, regardless of serum creatinine concentration
-*Poison*: A xenobiotic (exogenous chemical, including medications and drugs) or an endogenously found chemical (e.g., iron, copper, vitamins) resulting from exogenous exposure with the potential to cause toxicity.
-*Poisoning*: Exposure to a poison causing or capable of causing toxicity, regardless of intent. It includes intoxication, toxicity, and overdose
-*Refractory bradycardia*: per age-related defined standards, after vasopressor/inotropic support
-*Refractory hypotension*: per age-related defined standards, after adequate fluid challenge and vasopressor/inotropic support
-*Serious complications of catheter insertion*: these include hemothorax, pneumothorax, hemomediastinum, hydromediastinum, hydrothorax, subcutaneous emphysema retroperitoneal hemorrhage, embolism, nerve injury, arteriovenous fistula, tamponade, and death if occurring within 72 hours of insertion. Hematoma and arterial puncture were judged not serious and thus excluded from this composite outcome. DVT and infectious complications were not included considering the short duration of catheter use.
-*Serious complications of ECTR*: for hemodialysis and CKRT, these include air emboli and shock; minor bleeding from heparin, transient hypotension, and electrolytes imbalance were judged not serious. For hemoperfusion: these include severe thrombocytopenia, major bleeding, and hemolysis; transient hypotension, hypoglycemia, hypocalcemia, and thrombocytopenia were judged not serious. For therapeutic plasma exchange, these include citrate toxicity, severe allergic reaction, arrhythmia, and vasovagal reaction; hypotension, hypocalcemia, and urticaria were judged as not serious. They were attributed to the ECTR if occurring within 72 hours of initiation.
-*Severe poisoning*: Exposure to a poison causing or capable of causing, if left untreated, end-organ damage.

-*Shock or end-organ compromise*: Hypotension (systolic blood pressure < 90 mmHg or mean blood pressure < 65 mmHg) with the presence of cellular ischemia as evidenced by increased lactate concentration, acute kidney injury (AKI) as defined by the Kidney Disease Improving Global Outcomes (KDIGO) guideline, increased troponin, altered mental status, or decreased capillary refill

References

1. Institute of Medicine Committee on Standards for Developing Trustworthy Clinical Practice G. In: Graham R, Mancher M, Miller Wolman D, Greenfield S*, et al.* (eds). *Clinical Practice Guidelines We Can Trust*. National Academies Press (US): Washington (DC), 2011.

2. Ghannoum M, Nolin TD, Lavergne V*, et al.* Blood purification in toxicology: nephrology's ugly duckling. *Adv Chronic Kidney Dis* 2011; **18:** 160-166.

3. Lavergne V, Nolin TD, Hoffman RS*, et al.* The EXTRIP (Extracorporeal Treatments In Poisoning) workgroup: Guideline methodology. *Clin Toxicol* 2012; **50:** 403-413.

4. Bouchard J, Lavergne V, Roberts DM*, et al.* Availability and cost of extracorporeal treatments for poisonings and other emergency indications: a worldwide survey. *Nephrol Dial Transplant* 2017; **32:** 699-706.

5. Moher D, Shamseer L, Clarke M*, et al.* Preferred reporting items for systematic review and meta-analysis protocols (PRISMA-P) 2015 statement. *Syst Rev* 2015; **4:** 1.

6. Higgins JPT, Thomas J, Chandler J. *Cochrane Handbook for Systematic Reviews of Interventions*, 2nd edn. John Wiley & Sons: Chichester (UK), 2019.

7. Sterne JA, Hernan MA, Reeves BC*, et al.* ROBINS-I: a tool for assessing risk of bias in non-randomised studies of interventions. *BMJ* 2016; **355:** i4919.

8. Guyatt GH, Oxman AD, Vist GE*, et al.* GRADE: an emerging consensus on rating quality of evidence and strength of recommendations. *BMJ* 2008; **336:** 924-926.

9. Schunemann HJ, Oxman AG: Handbook for grading the quality of evidence and the strength of recommendations using the GRADE approach. In (vol 2020), Hamilton, ONT, 2020
